# Effects of heavy metal exposure on oral microbial communities in women with different menopausal status

**DOI:** 10.1128/spectrum.03123-25

**Published:** 2026-03-23

**Authors:** Keke Yang, Zhen Yang, Huan Li, Hongling Liu, Susu Cao, Yuanyuan Bao, Lu Feng, Li Zhang, Jingping Niu, Tian Tian

**Affiliations:** 1 School of Public Health, Lanzhou University 628733, Lanzhou, China; 2 Lanzhou Maternal and Child Health Care Hospital, Lanzhou, China; 3 School of Stomatology, Lanzhou University 12426, Lanzhou, China; Nova Southeastern University, Fort Lauderdale, Florida, USA

**Keywords:** bacterial oral microbiome, buccal mucosa, heavy metals, menopause, NGS (next-generation sequencing)

## Abstract

This study investigates the effects of long-term heavy metal exposure on the oral mucosal microbiota in women. By measuring both external environmental heavy metal exposure and internal heavy metal exposure indicators in the human body, it aims to elucidate the complex interactions between external environmental exposure and internal exposure, as well as their potential association with menopausal status. We analyzed oral microbial composition, function, and co-occurrence networks in 47 women from polluted and control areas. Heavy metal exposure significantly altered oral microbial diversity and functional pathways, with molybdenum (Mo) exhibiting a uniquely strong influence. Key metabolic pathways related to cardiovascular disease and carbohydrate metabolism were enriched in specific groups. Network analysis revealed a loss of keystone species and structural simplification in postmenopausal women from the polluted area. These findings suggest that heavy metal exposure alters oral microbial communities, and these alterations correlate with shifts in host metabolic pathways that are known to be associated with menopausal hormonal changes, potentially impacting women’s health during this transition.

**IMPORTANCE:**

This study reveals, for the first time, how chronic heavy metal exposure and menopause interact to disrupt the female oral microbiome. We identify Mo as a key metal, correlating strongly with specific bacteria and linked to downregulated cardiovascular and metabolic pathways. Critically, postmenopausal women in polluted areas exhibit a severe loss of keystone species and a collapsed microbial network structure. These findings position the dysregulated oral microbiome as a potential mechanistic link between environmental metal exposure and the heightened systemic risks—such as metabolic disorders and chronic inflammation—observed in postmenopausal women, highlighting new targets for preventive health strategies.

## INTRODUCTION

The human oral microbiota is highly diverse and has a complex ecology that includes bacteria, microeukaryotes, archaea, and viruses. Among these microorganisms, the most widely studied are bacteria. In terms of species, the oral microbiome stands out as one of the most important and complex microbial communities in human physiology, with the oral microbiome being second only to the colon in complexity (1). The oral microbiota is present in saliva and on the surface areas of the oral cavity (such as supragingival plaque, subgingival plaque, tongue surface, and periodontal pockets). Studies of the human oral microbiome have shown that microorganisms residing in the oral cavity play an important role in the overall health of the host, and dysbiosis of the oral microbiome is frequently associated with the pathogenesis of oral and systemic diseases (2).

With the development of cities, non-ferrous metals play an important role in boosting the national economy; however, the environmental impacts of heavy metals emitted during the production of non-ferrous metals are of increasing concern worldwide (3). Rapid urbanization accelerates the release of anthropogenic heavy metals from localities into wider water and agricultural systems, which further cause enrichment in the food chain through, for example, soil and groundwater (4–6), posing a serious threat to ecosystems and public health (7). In particular, areas around mining and smelting plants are often contaminated with heavy metals, ultimately causing irreversible ecological damage (8). The accumulation of heavy metals in agricultural soils not only leads to the depletion and degradation of soil resources but also enters the human body through the soil-food-human food chain and accumulates in agricultural products and the human body through biomagnification, affecting the safety of agricultural products and human health. Once these metals are over-accumulated and enriched in the human body, they can disrupt normal biochemical reactions for a long time, causing carcinogenic effects, affecting key organs, and ultimately destroying normal biological functions (9, 10). Studies have shown that nickel (Ni) has the greatest effect on the skin, causing pimples, herpes, and erythema, followed by cancer, neurological disorders, systemic diseases, and reduced fertility. Copper (Cu) can lead to chronic toxicity manifested by neurological weakness, metabolic disorders, cellular carcinogenesis, and damage to brain tissues. Zinc (Zn), a trace element in the human body, is a cause of disease when it is too high or too low (11). Zn deficiency can lead to skin damage, neurological disorders, and decreased immunity, and excess Zn can lead to zinc toxicity and symptoms such as vomiting and diarrhea (12). Internal exposure biomarkers are commonly used to assess the level of environmental heavy metal exposure in human exposure, and whole blood indicators are good biomarkers for a wide range of toxic metals. For example, blood cadmium (Cd), blood Zn, and blood Ni are commonly used to assess recent exposure (13). The vast majority of heavy metals in the human body, lead (Pb), can enter the blood cells and be transported by the blood to the bone tissue, where it replaces calcium (Ca) and binds to phosphates, forming a stable complex that replaces calcium phosphate salts in the bone matrix (13). The metal mercury (Hg) enters the erythrocytes in the blood, accumulates in the erythrocytes, binds to hemoglobin, and can also readily cross the blood-brain barrier and enter the central nervous system (13). Therefore, blood Pb and blood Hg can be used as internal exposure biomarkers commonly used for long-term exposure.

Menopause is characterized by cessation of menstruation due to the termination of ovarian function, which can be complete atrophy with severe estrogen deficiency (14). The oral mucosa contains estrogen receptors, and changes in hormone levels can directly affect the oral environment. Although menopause is not considered a major risk factor for the development of periodontitis, it has been suggested that it contributes to the severity of the disease (15). Many menopausal women suffer from oral discomfort, dry mouth, and tooth loss in addition to menopausal symptoms (16). Therefore, it may be clinically relevant to understand the effects of sex hormone changes in women during menopause on the characterization of the oral microbiota.

In general, oral microorganisms in healthy individuals are in a state of dynamic equilibrium. The oral bacterial community is mainly composed of phylum Actinobacteria, Bacteroidetes, Fusobacteria, Firmicutes, and Proteobacteria (17). The oral microbiome is susceptible to environmental influences such as antibiotics, disease states, diet, and smoking (17, 18). Loss and increase in species diversity affect ecological stability (19) and the sustainability of ecosystem functions and services (20). Evidence suggests that environmental changes enhance microbial interactions, which leads to changes in microbiome diversity, further affecting microbial community stability (21). Microbial communities with higher diversity tend to recover faster after environmental damage and disturbance, implying that those with higher biodiversity show better resilience in response to environmental disturbance (22). In the oral cavity, a variety of endogenous and exogenous factors can alter the relative abundance and composition of the oral microbial microbiome, leading to microbial dysbiosis (17) and the development of diseases such as oral caries, periodontitis, and even systemic diseases (23). Decreases in oral microbial diversity have been associated with the development of oral diseases (24). It has been demonstrated that prolonged exposure to a wide range of metals disrupts the normal bacterial community in the human oral cavity (25, 26) and alters bacterial function and diversity, even at low levels of heavy metal content (27). In addition, menopausal women experience a rapid decline in estrogen levels in conjunction with menopause. Estrogen has beneficial effects on the upper and lower respiratory tracts, increasing nasal mucus, which contains lactoferrin, IgA, IgG, and mucin estrogen with antimicrobial properties (28). Estrogen promotes enhancement of the innate and adaptive immune system, increasing macrophage, dendritic cell, and natural killer cell activity, as well as inflammatory cytokines (e.g., IL-1, TNF-α, and IL-6), and promotion of type I interferon responses (29, 30). Therefore, the decline in estrogen in postmenopausal women affects respiratory health. Other studies have demonstrated that postmenopausal women have a worse oral status than premenopausal women, with high levels of oral proinflammatory cytokines, which may be related to estrogen levels (30, 31). *Prevotella* has been implicated as a causative agent of altered oral health in pre- and post-menopausal women (30, 32, 33), suggesting that altered oral microecological balance affects women’s oral health at the time of menopause.

This study aims to elucidate the effects of heavy metal exposure on the composition of the oral microbiome and its association with menopausal status. Characterizing these two factors that influence the composition and functional characteristics of the oral microbiome will help us understand the differences in the oral microbiome and its importance in regulating women’s health.

## RESULTS

### Heavy metal pollution in the study area

The results of heavy metal concentrations in soil from polluted and non-polluted areas showed that in the polluted soil, our results indicated that the mean values of six metals (Pb, Cd, Sb, Zn, Hg, and Cu) were significantly higher than in the control area (*P* < 0.05; Table S1). Blood concentrations of both metals (Cd and Pb) were significantly higher in subjects living in the contaminated area than in the control area (*P* < 0.001; Table S2). Compared to the general population reference range, Group H exhibited significantly higher exposure levels to Cd, Pb, and Mn, while Ni exposure was elevated in both groups (Table S2).

### Description of the study population

The study included a total of 47 women. Among them, 29 were menopausal women (61.7%) with a mean age of 55.9 years, and 18 non-menopausal women (38.3%) with a mean age of 52.7 years (Table 1).

**TABLE 1:** Baseline characteristics of the study population

Group	NM	HM	NN	HN	*P*-value
Age [years]	53.5 ± 5.6	57.4 ± 5.8	53.4 ± 3.1	52.4 ± 5.6	0.127
Age at Menarche [years]	16.4 ± 2.1	16.5 ± 2.3	15.8 ± 0.8	15.3 ± 1.4	0.134
Number of pregnancies	2.1 ± 0.8	2.8 ± 1.0	2.0 ± 0	2.5 ± 0.6	0.134
Number of births	1.9 ± 0.5	2.4 ± 0.7	2.0 ± 0	2.4 ± 0.6	0.396
Smoking	0 (0.0%)	0 (0.0%)	0 (0.0%)	0 (0.0%)	–^*a*^
Alcohol consumption	0 (0.0%)	0 (0.0%)	0 (0.0%)	0 (0.0%)	–
Meal structure (Three meals a day / Two meals a day)	5/6	14/4	2/3	10/3	0.146
Number of persons	11	18	5	13	–

^*a*^ “–” indicates that a* P*-value is not applicable.

### Alpha- and beta-diversity analysis of oral microbiota

**Fig 1:**
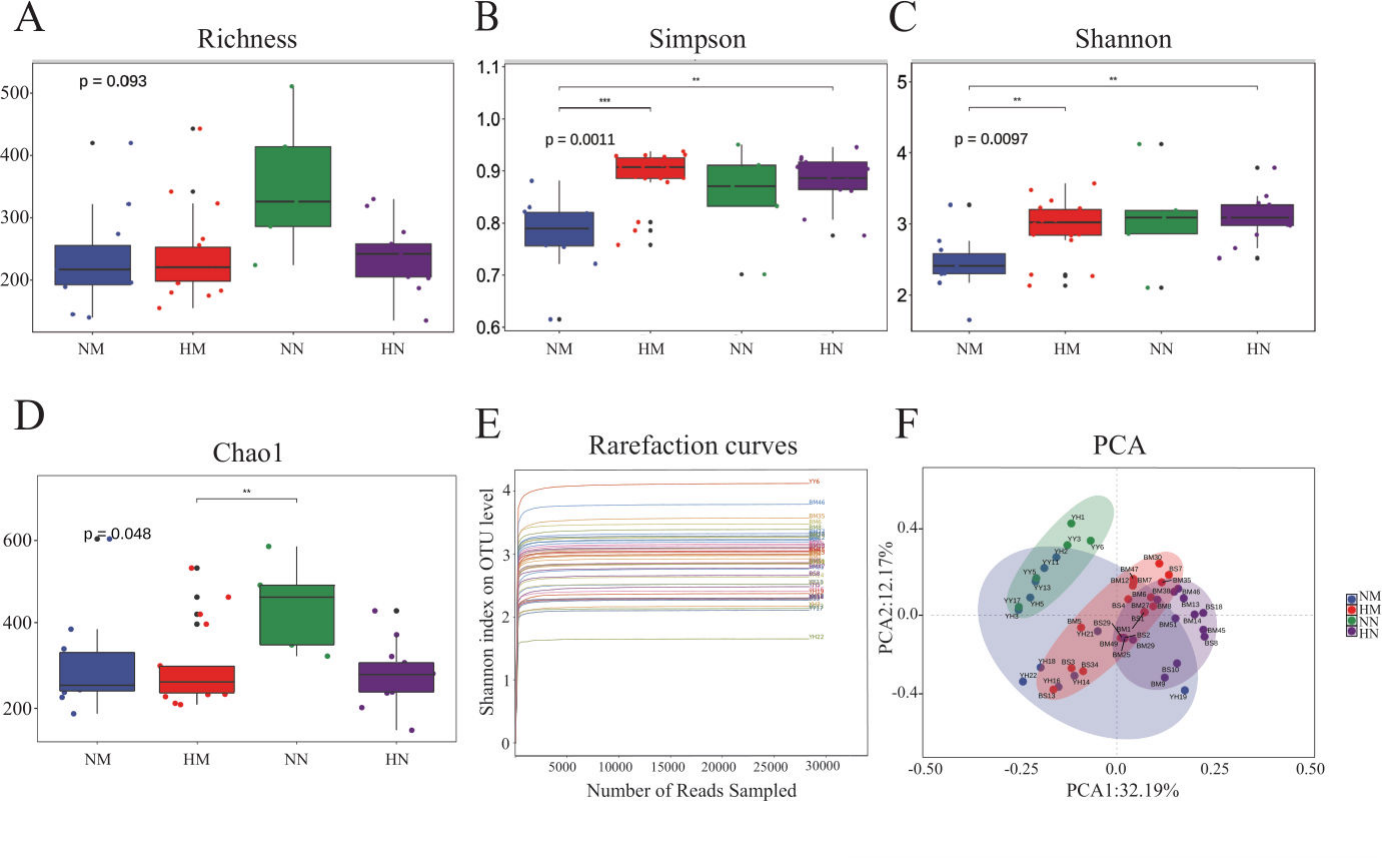
Analysis of α-diversity and principal coordinate analysis of buccal mucosal bacterial communities. (**A-D**) Differences in bacterial α diversity indices between the NM, HM, NN, and HN groups. Significant differences were determined by using the Wilcoxon rank-sum test. (*P < 0.05; **P < 0.01; ***P < 0.001). (**E**) Rarefaction curves for the bacterial community data set. The flat curve indicated that the sequence number used for analyses was adequate. (**F**) Principal component analysis (PCA) of bacterial community dissimilarities. Significant differences in bacterial β diversity between the four groups: group NM (non-heavy metal-exposed and post-menopausal), group HM (heavy metal-exposed and post-menopausal), group NN (non-heavy metal-exposed and non-menopausal), and group HN (heavy metal-exposed and non-menopausal).

The dilution curve of the samples indicates that the sequencing data accurately reflects the diversity of the oral bacterial community (Fig. 1E).

We found that the Richness and Shannon indices were the largest in the NN group, indicating that microbial relative abundance and diversity were the highest in this group; the Simpson indices showed significant differences between the NM and heavy metal (HM) groups, and between the NM and HN groups (*P* < 0.05). Chao1 indices showed that there were significant differences between the HM group and the NN group (Fig. 1A through D, *P* < 0.05). These results suggest that heavy metal exposure and menopausal status affect the alpha diversity of oral bacterial communities.

Significant differences were observed in the microbial structures of oral samples across the different groups (*P* < 0.05; Fig. 1F and Table 2). The greatest differences between microbial communities were found between the NM and HN groups (R^2^ = 0.428, *P* < 0.01).

**TABLE 2:** PERMANOVA analysis of intergroup differences in microbial communities^*a*^

ANOVA	*P*.Value	Sig
Overall four groups	0.001	***
NM vs. HM	0.0012	**
NM vs. NN	0.042	*
NM vs. HN	0.0012	**
HM vs. NN	0.0012	**
HM vs. HN	0.0012	**
NN vs. HN	0.0012	**

^*a*^ Note: *P < 0.05, **P < 0.01, and ***P < 0.001.

### Bacterial community structure of oral samples

**Fig 2:**
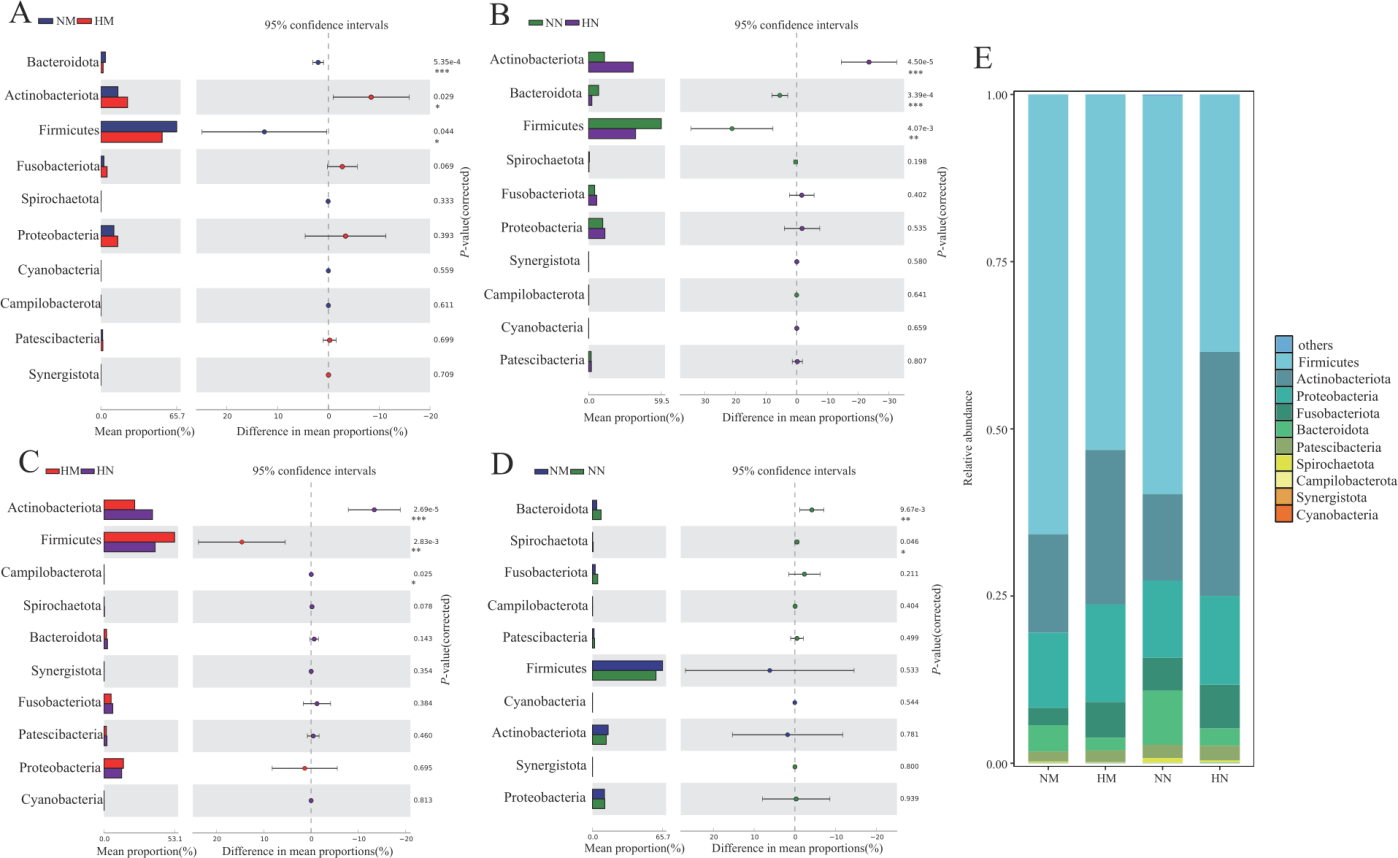
The bacterial community composition of the NM, HM, NN, and HN groups (**E**). Compositional differences in bacterial communities of the buccal mucosa at the phylum (NM, HM, NN, and HN groups) (**A-D**). The *P*-value was calculated using the Wilcoxon rank-sum test and adjusted by using the false discovery rate. (*P < 0.05; **P < 0.01; ***P < 0.001） group NM (non-heavy metal-exposed and post-menopausal), group HM (heavy metal-exposed and post-menopausal), group NN (non-heavy metal-exposed and non-menopausal), and group HN (heavy metal-exposed and non-menopausal).

At the phylum level, a total of 39 phyla were detected, and the phyla with relative abundance > 1% were Firmicutes, Actinobacteriota, Proteobacteria, Fusobacteria, Bacteroidota, and Patescibacteria. These six phyla accounted for more than 95% of the oral microbiota of all samples, which are the main dominant phyla in human oral microbiota (Fig. 2A and B). In the heavy metal-exposed groups (HM and HN), the relative abundance of Actinobacteriota was significantly higher than that in the control groups (NM and NN) (*P* < 0.05); in the menopausal groups (NM and HM), the relative abundance of Firmicutes was significantly higher than that in the non-menopausal group (NN and HN) (*P* < 0.01).

**Fig 3:**
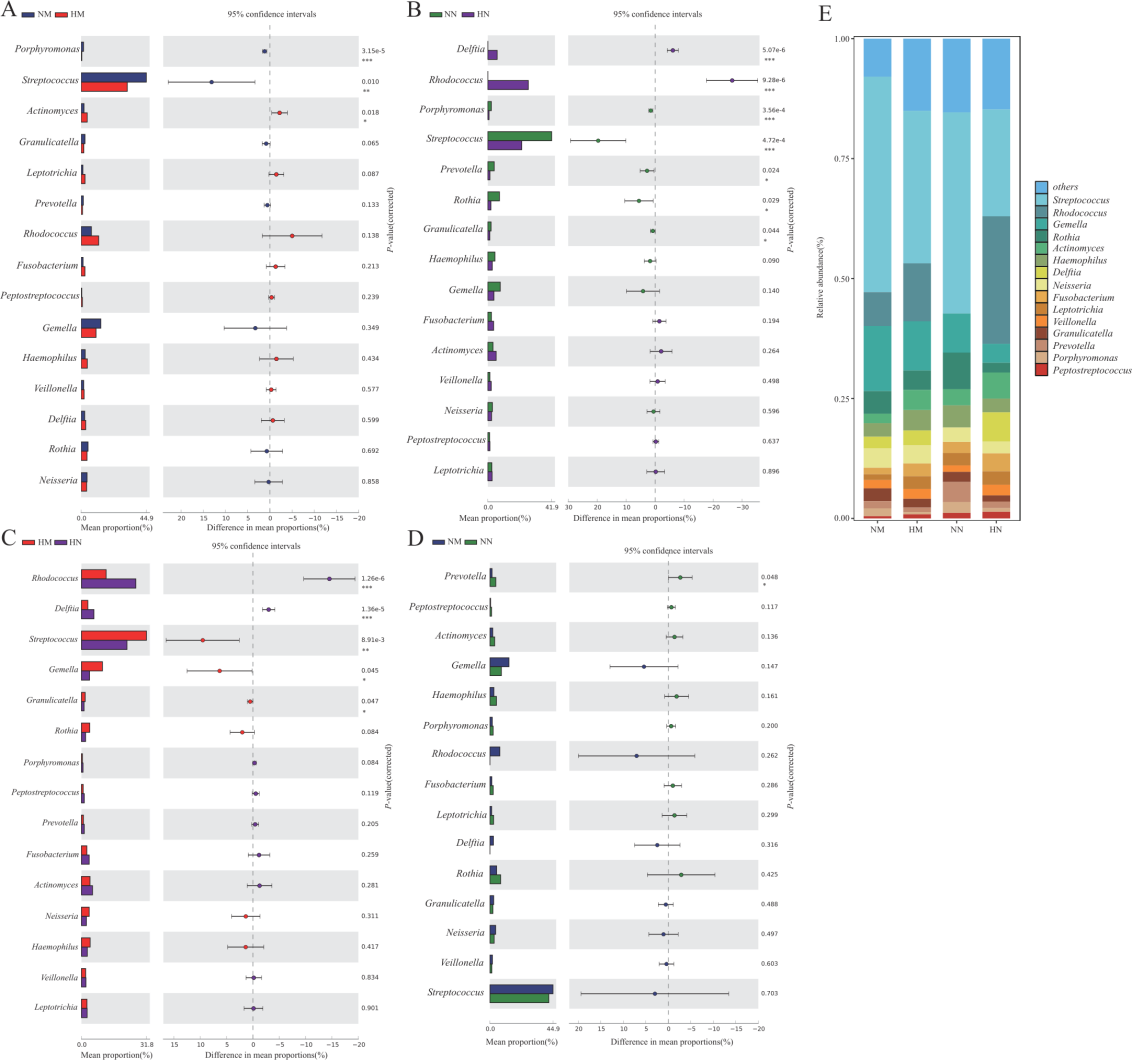
The bacterial community composition of the NM, HM, NN, and HN groups (**E**). Compositional differences in bacterial communities of the buccal mucosa at the genus (**A-D**) levels. The *P* value was calculated using the Wilcoxon rank-sum test and adjusted by using the false discovery rate. (*P < 0.05; **P < 0.01; ***P < 0.001） group NM (non-heavy metal-exposed and post-menopausal), group HM (heavy metal-exposed and post-menopausal), group NN (non-heavy metal-exposed and non-menopausal), and group HN (heavy metal-exposed and non-menopausal).

At the genus level, a total of 901 genera were detected, with relative abundance > 1% being *Actinomyces*, *Delftia*, *Fusobacterium*, *Gemella*, *Granulicatella*, *Haemophilus*, *Leptotrichia*, *Neisseria*, *Peptostreptococcus*, *Porphyromonas*, *Prevotella*, *Rhodococcus*, *Rothia*, *Streptococcus*, and *Veillonella* (Fig. 3E). Among them, the relative abundance of *Actinomyces*, *Delftia*, *Leptotrichia*, *Fusobacterium,* and *Rhodococcus* was significantly higher in the heavy metal exposed groups (HM and HN) than in the control group (NM and NN) (Fig. 3A and B, *P* < 0.05), whereas *Porphyromonas*, *Streptococcus*, *Granulicatella*, *Prevotella,* and *Rothia* in the control group were significantly higher than those in the exposed group (*P* < 0.05); in the menopausal group (NM and HM), the relative abundance of *Streptococcus*, *Gemella* and *Granulicatella* were significantly higher than that in the non-menopausal groups (NN and HN) (Fig. 3C and D, *P* < 0.01), while *Fusobacterium*, *Prevotella*, *Rhodococcus,* and *Delftia* were significantly enriched in the non-menopausal groups (*P* < 0.05).

Seven genera were found to be significantly different between the NM and HM groups: *Porphyromonas* and *Streptococcus* were enriched in the NM group, and *Rhodococcus*, *Actinomyces*, *Eptotrichia*, *Fusobacterium*, and *Delftia* were found to be significantly enriched in the HM group. Five genera were significantly different between the NN and HN groups: *Porphyromonas* and *Streptococcus* were enriched in the NN group, which was consistent with the NM group, and *Rhodococcus*, *Delftia,* and *Prevotella* were enriched in the HN group. Among them, the relative abundance of *Rhodococcus* and *Delftia* was significantly (*P* < 0.05) higher in the HM and HN groups, *Porphyromonas* and *Streptococcus* was significantly (*P* < 0.05) higher in the NM and NN groups; two genera with higher abundance were found to be significantly different between the NM and NN groups: *Prevotella* and *Fusobacterium* were enriched in the NN group; five genera with higher abundance were found to be significantly different between the HM and HN groups: *Gemella* and *Streptococcus* were enriched in the HM group, while *Rhodococcus*, *Delftia*, and *Fusobacterium* were enriched in the HN group. The relative abundance of *Fusobacterium* was significantly (*P* < 0.05) higher in NN and HN groups, *Prevotella* was significantly (*P* < 0.05) higher in the NN group, and *Streptococcus* and *Gemella* were significantly (*P* < 0.05) higher in the HM group.

### Predictive function analysis using KEGG

**Fig 4:**
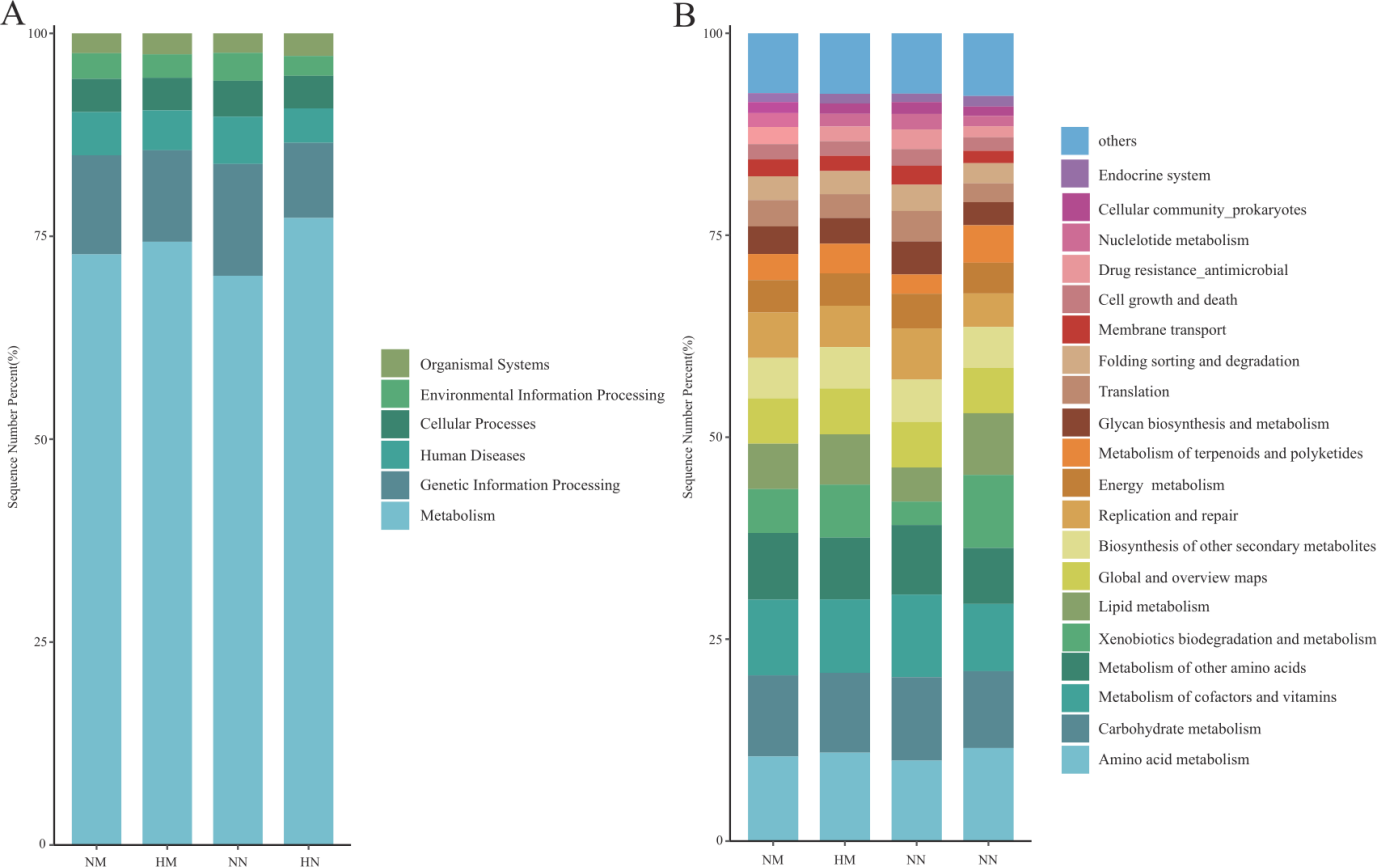
Histogram of the distribution of KEGG level 1 functional pathway genes (**A**) and level 2 functional pathway genes (**B**) among different groups. Group NM (non-heavy metal-exposed and post-menopausal), group HM (heavy metal-exposed and post-menopausal), group NN (non-heavy metal-exposed and non-menopausal), and group HN (heavy metal-exposed and non-menopausal).

**Fig 5:**
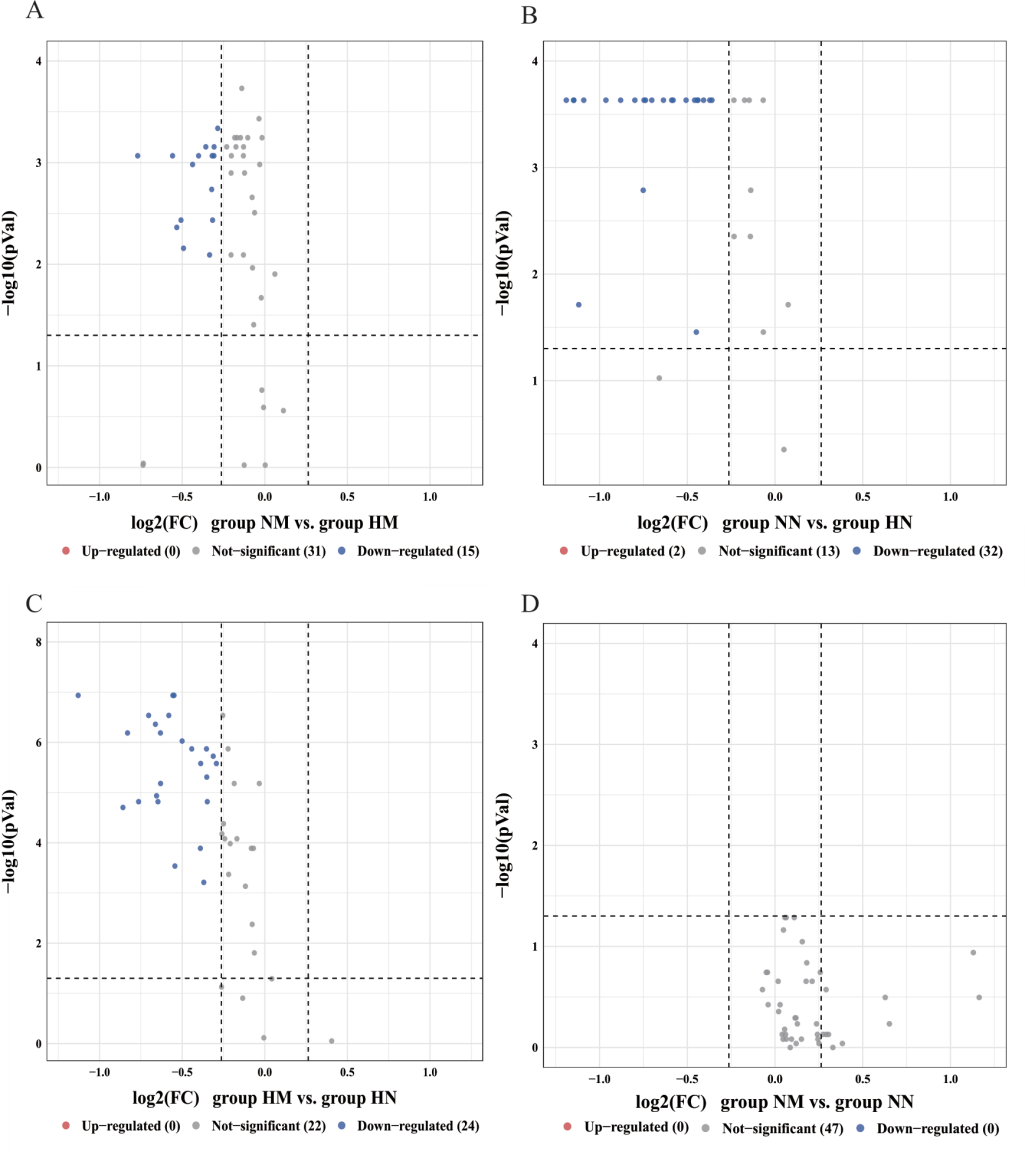
Volcano plots of differences in relative abundance of functional genes of secondary pathways in oral bacterial communities. (**A**) NM vs. HM, (**B**) NN vs. HN, (**C**) HM vs. HN, and (**D**) NM vs. NN. FC denotes fold difference. Red, blue, and gray circles indicate upregulated, downregulated, and non-significantly different genes. Group NM (non-heavy metal-exposed and post-menopausal), group HM (heavy metal-exposed and post-menopausal), group NN (non-heavy metal-exposed and non-menopausal), and group HN (heavy metal-exposed and non-menopausal).

**Fig 6:**
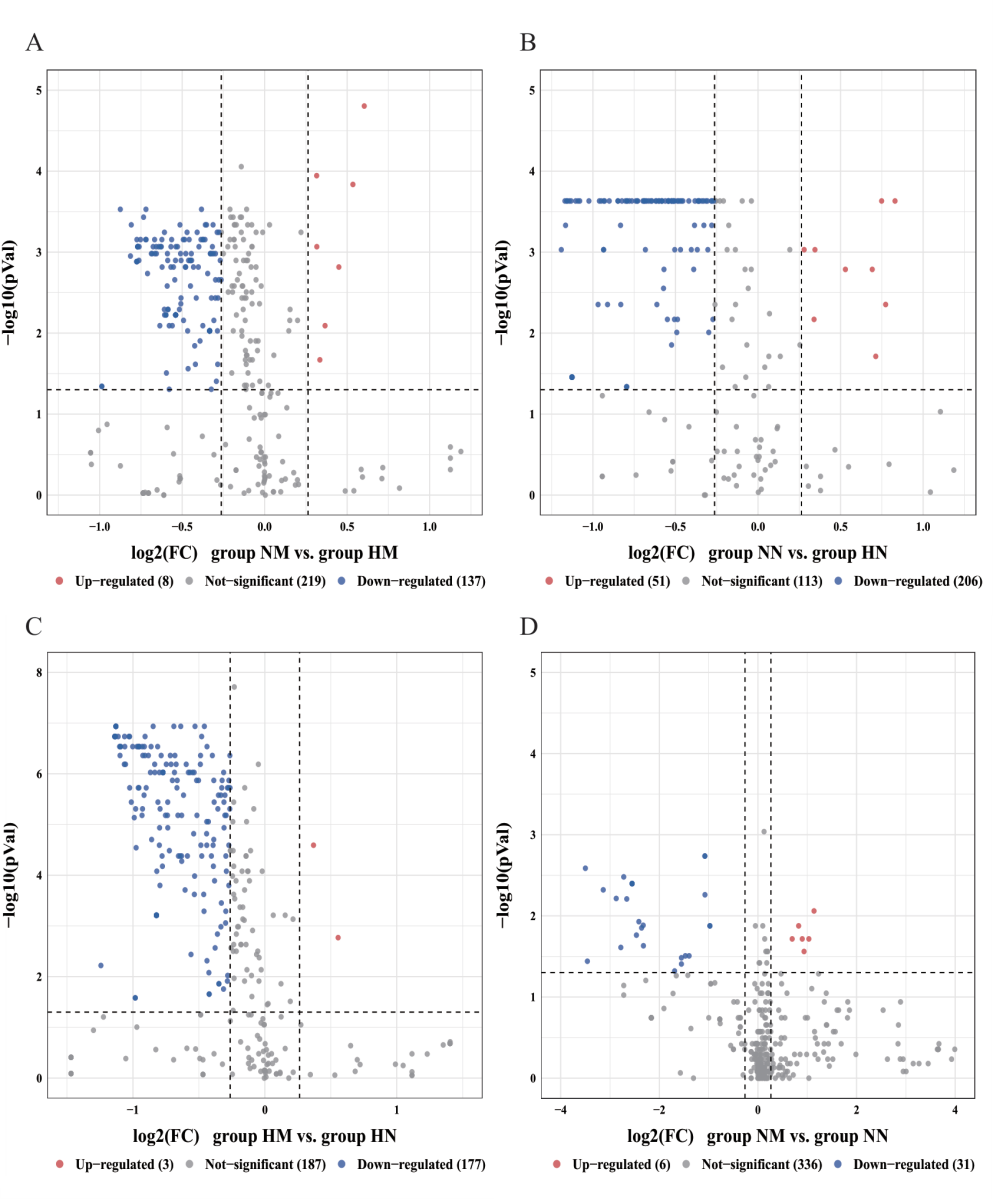
Volcano plots of differences in relative abundance of functional genes of the tertiary pathway in oral bacterial communities. (**A**) NM vs. HM, (**B**) NN vs. HN, (**C**) HM vs. HN, and (**D**) NM vs. NN. FC denotes the multiplicity of differences. Red, blue, and gray circles indicate upregulated, downregulated, and non-significantly different genes. Group NM (non-heavy metal-exposed and post-menopausal), group HM (heavy metal-exposed and post-menopausal), group NN (non-heavy metal-exposed and non-menopausal), and group HN (heavy metal-exposed and non-menopausal).

Differences in microbial gene function in terms of metabolic pathways between the four groups were assessed using KEGG enrichment analysis. Level 1 KEGG pathways mainly involved six functional pathways, among which the most abundant pathway was metabolism, with an average relative abundance of 73.62% of the annotated genes (Fig. 4A). In secondary pathways (Fig. 4B), the HM exposure group exhibited a widespread decrease in the relative abundance of 15 functional genes (Fig. 5A), with significant reductions observed in the cardiovascular disease and substance-dependence pathways. At the tertiary pathway level, key pathways such as cardiac muscle contraction and the insulin signaling pathway were suppressed (Fig. 6A; Tables S3 and S6). This indicates that heavy metal exposure may impair host cardiac muscle function and glucose homeostasis regulation, serving as a core factor driving increased cardiovascular disease and diabetes risk. In non-menopausal women, 32 functional genes showed widespread relative abundance reduction (Fig. 5B), involving immunity, cancer, and various basal metabolic pathways (Tables S4 and S7). Notably, the PPAR signaling pathway regulating glucose and lipid metabolism was significantly suppressed. This suggests that heavy metal exposure may induce broader metabolic dysregulation and immune dysfunction in reproductive-age women, laying the groundwork for subsequent metabolic syndrome development. Under identical heavy metal exposure conditions, postmenopausal women (HM) exhibited more pronounced relative abundance declines than premenopausal women (HN), involving 24 secondary pathways, including substance dependence and nervous system (Fig. 5C; Tables S5 and S8). This suggests that menopausal status may amplify the negative effects of heavy metal exposure on neurological and mental health (e.g., addiction and neurodegeneration), creating a double blow. Additionally, the microbial functional changes induced by menopausal status itself were relatively limited (Fig. 5D; Table S9).

### Correlation analysis between oral microorganisms and blood heavy metals

**Fig 7:**
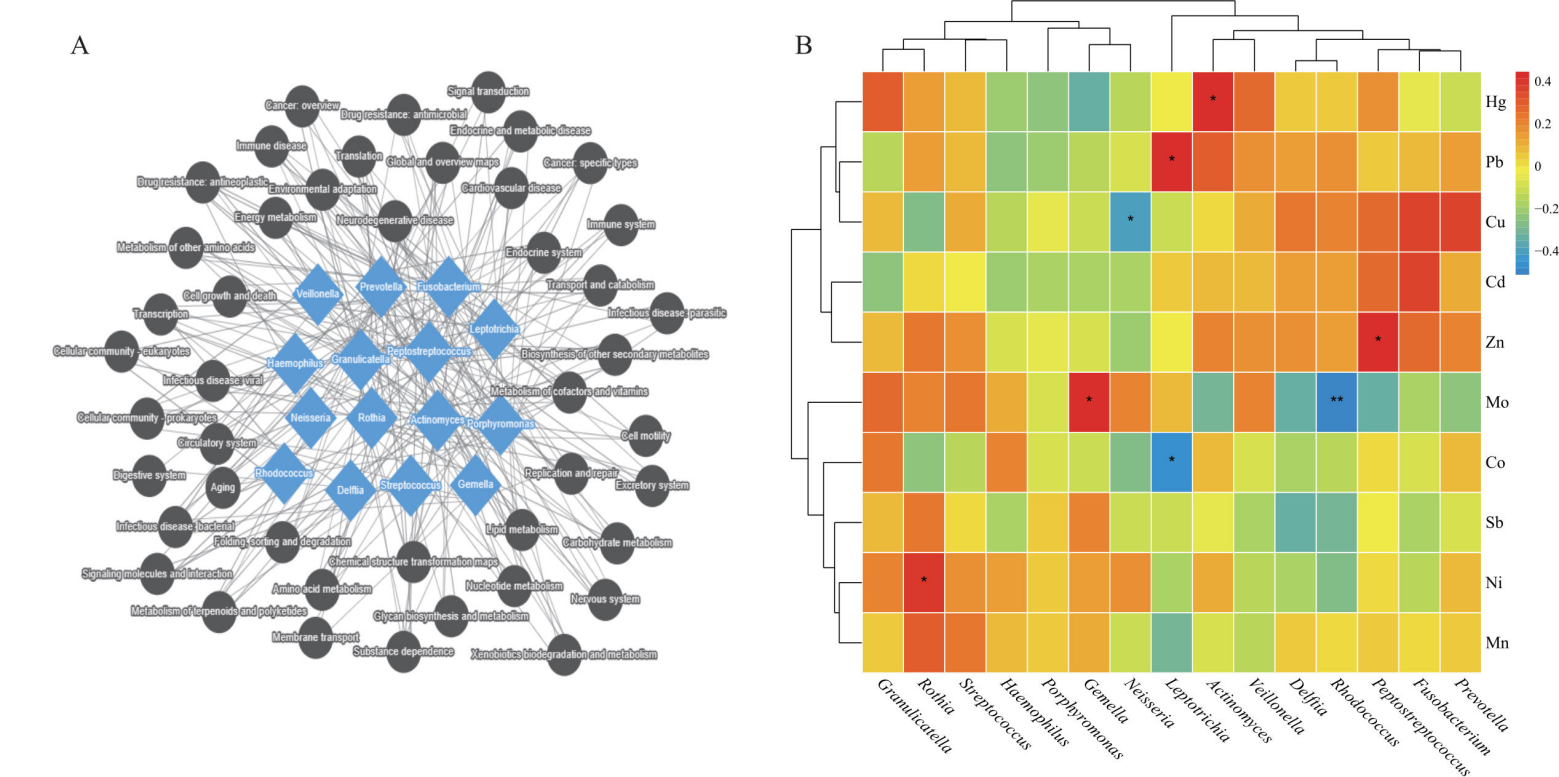
(**A**) Net for correlation analysis of differential bacteria with differential functional pathways. (**B**) Correlation between genus level bacteria and blood heavy metals (*P < 0.05, **P < 0.01, ***P < 0.001; *P*-values calculated using the SparCC algorithm). Group NM (non-heavy metal-exposed and post-menopausal), group HM (heavy metal-exposed and post-menopausal), group NN (non-heavy metal-exposed and non-menopausal), and group HN (heavy metal-exposed and non-menopausal).

The correlation between blood heavy metal concentrations and the relative abundance of bacteria in the buccal mucosa showed that ten heavy metals (Cd, Pb, Zn, Cu, Ni, Mn, Mo, Co, Hg, and Sb) had a significant effect on the bacterial community (Fig. 7B). *Actinomyces*, which had a significant increase in relative abundance in the heavy metal exposed groups (HM, HN), was positively correlated with Hg (*P* < 0.05), and *Peptostreptococcus*, which had a relatively high abundance, was positively correlated with Zn (*P* < 0.05). In the menopausal groups (NM and HM), the genera *Gemella* and *Rothia,* with higher relative abundance, were positively correlated with Mo (*P* < 0.05) and Ni (*P* < 0.05), and *Rhodococcus* and *Leptotrichia,* with lower relative abundance, were negatively correlated with blood concentrations of Mo (*P* < 0.01) and Co (*P* < 0.05).

### Correlation analysis of differential bacteria with differential function pathways

**Fig 8:**
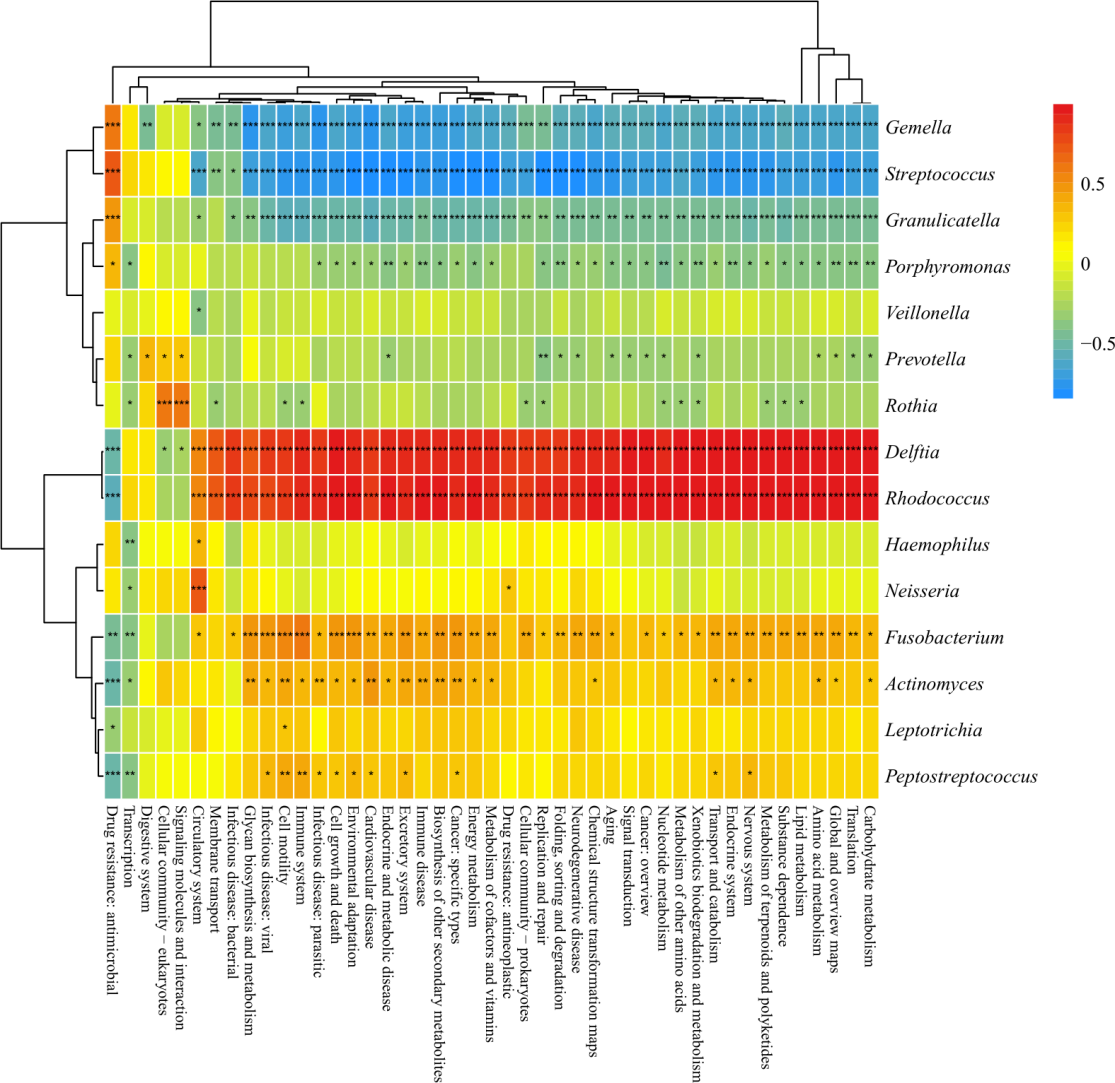
Heatmap for correlation analysis of differential bacteria with differential functional pathways (*P < 0.05, **P < 0.01, ***P < 0.001; *P*-values calculated using the SparCC algorithm). Group NM (non-heavy metal-exposed and post-menopausal), group HM (heavy metal-exposed and post-menopausal), group NN (non-heavy metal-exposed and non-menopausal), and group HN (heavy metal-exposed and non-menopausal).

To elucidate the relationship between differential bacteria and differential function, 15 dominant species at the genus level were correlated with 45 secondary differential functional pathways (Fig. 7A and 8). The results showed that *Delftia* and *Rhodococcus* were strongly positively correlated with all 41 functional pathways (*P* < 0.001); *Granulicatella*, *Gemella,* and *Streptococcus* were strongly negatively correlated with all 37 differential functional pathways (*P* < 0.01); *Fusobacterium* and *Actinomyces* were also positively correlated with more pathways and negatively correlated with drug resistance: antimicrobial and transcription (*P* < 0.05). Other dominant bacterial species are significantly correlated with secondary pathways (*P* < 0.05).

### Ecological network analysis of oral microbiota

**Fig 9:**
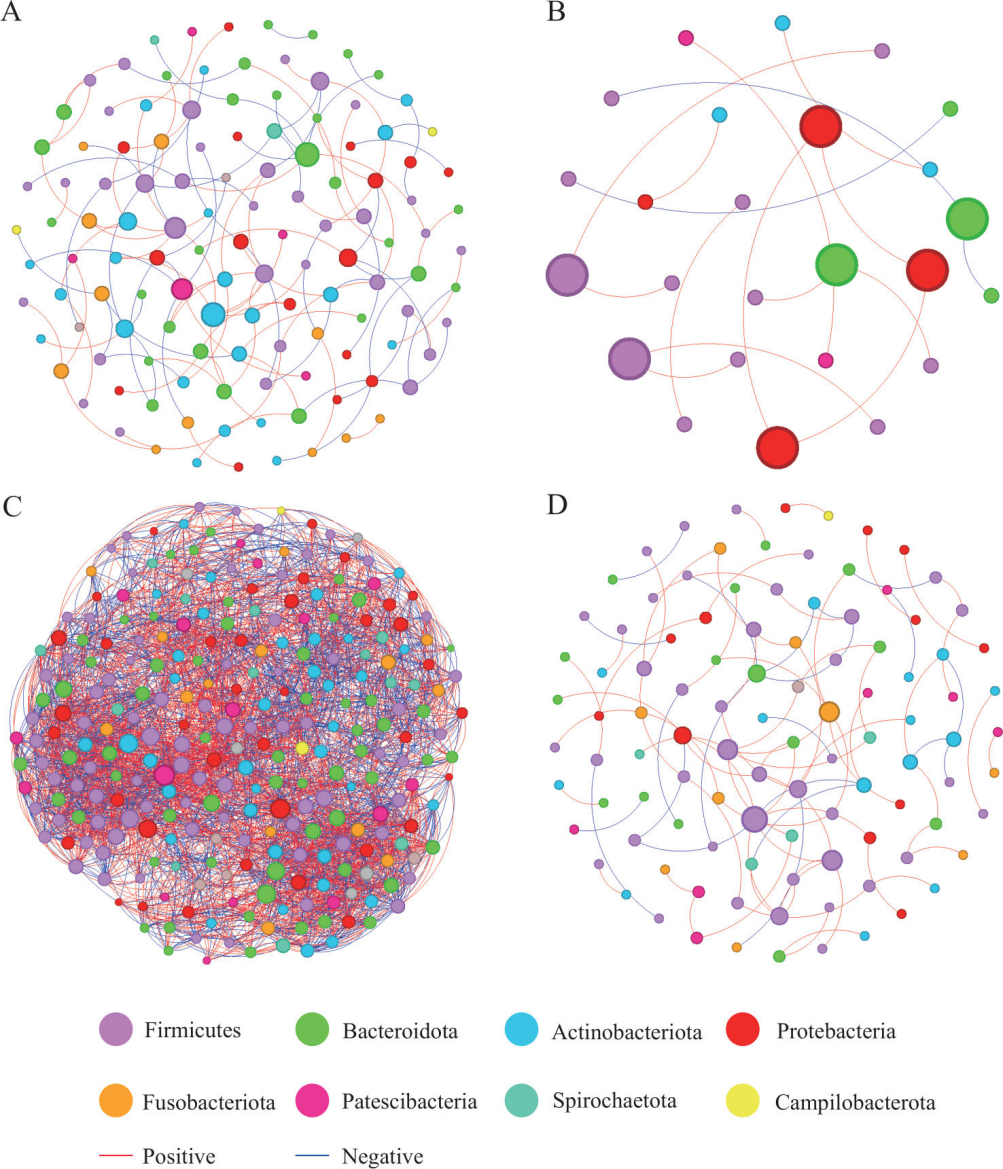
Molecular ecological networks were constructed based on the correlation between bacterial OTUs. The networks of group NM (**A**), group HM (**B**), group NN (**C**), and group HN (**D**) are shown. Node sizes are proportional to the number of connections. Each node represents a bacterial OTU and is colored according to its phylum-level classification relationship. Red lines indicate negative associations between bacterial OTUs, while blue lines indicate positive associations. Group NM (non-heavy metal-exposed and post-menopausal), group HM (heavy metal-exposed and post-menopausal), group NN (non-heavy metal-exposed and non-menopausal), and group HN (heavy metal-exposed and non-menopausal).

The overall network-level characteristics of the microbial networks were compared between the different exposure groups (Table 3). The co-occurrence networks of bacterial OTUs in the four groups are shown in Fig. 9.

**TABLE 3:** Network-level topological features of the bacterial subnetworks in the human oral

Group	NM	HM	NN	HN
Nodes	122	25	265	103
Edges	118	16	4180	90
RMT cutoff	0.77	0.77	0.77	0.77
Coefficient (avgCC)	0.037	0.120	0.453	0.043
Average path distance (GD)	8.896	1.200	2.278	3.179
Geodesic efficiency (E)	0.174	0.9	0.494	0.458
Harmonic geodesic distance (HD)	5.743	1.111	2.026	2.184
Average degree (avgK)	1.934	1.280	31.547	1.748
Graph density (D)	0.016	0.053	0.119	0.017
Modularity	0.836	0.882	0.420	0.862
pp	57.63%	81.25%	57.09%	75.56%
np	42.37%	18.75%	42.91%	24.44%

The NN group has the most network nodes and edges and has the highest avgCC, avgK, and D values, indicating that the NN group’s network pattern is more complex and stable, more robust than other groups, and more closely connected. Group HM exhibits the opposite trend, featuring the smallest network size and sparse connectivity. The proportion of positively associated edges was significantly greater than that of negatively associated edges across all four groups.

The network nodes of all groups were associated with eight different bacterial phyla. The relative abundance of Firmicutes was the highest in all group networks; Bacteroidota and Actinobacteriota had lower relative abundance in the network of the HM group than that of the NM group; Bacteroidota had higher relative abundance in the NN group than that of the HN group; and Proteobacteria had higher relative abundance in the network of the HM group than that of the HN group.

**Fig 10:**
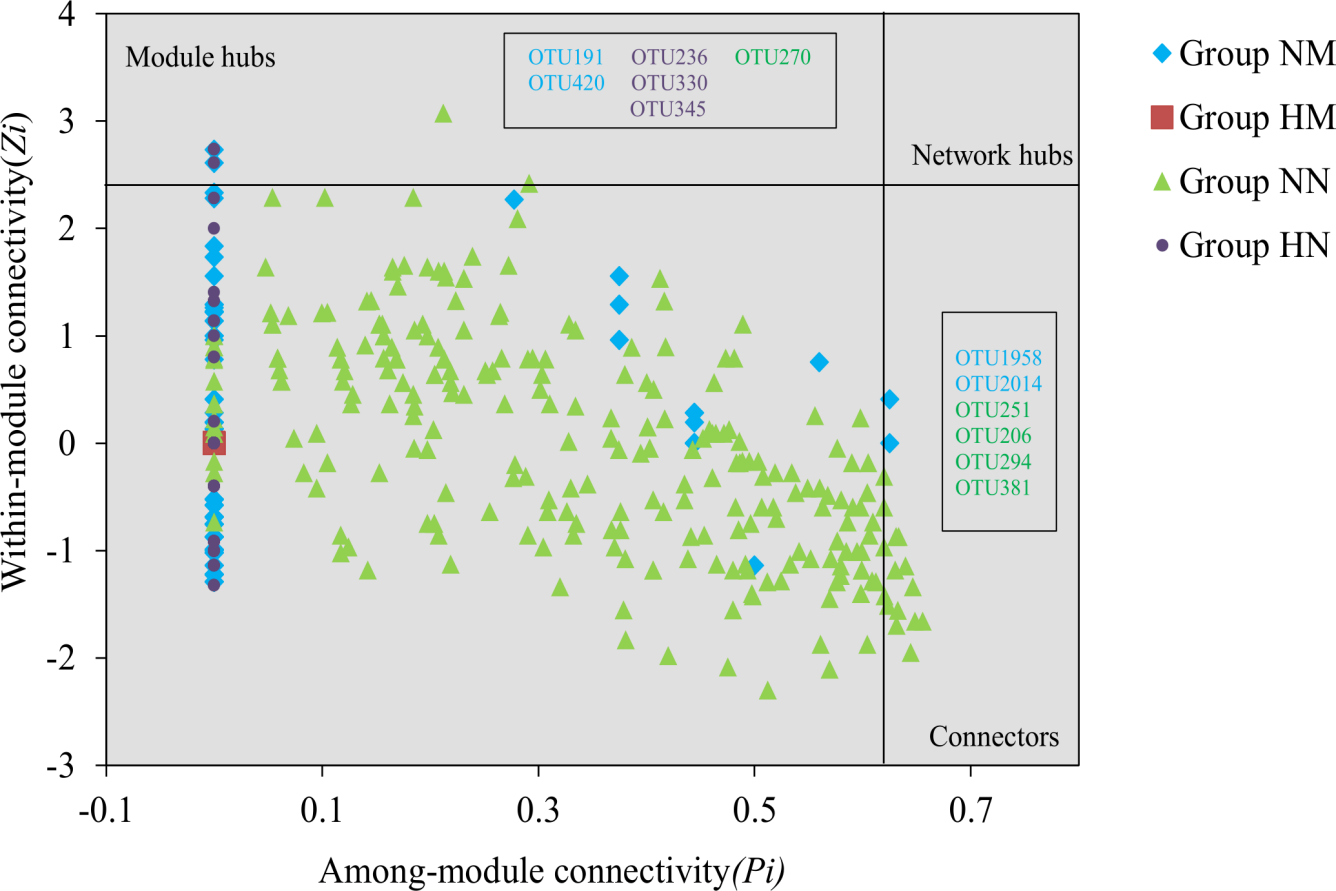
Topological roles of group NM, group HM, group NN, and group HN buccal mucosa bacteria co-occurring network nodes. Boxed are keystone species. Group NM (non-heavy metal-exposed and post-menopausal), group HM (heavy metal-exposed and post-menopausal), group NN (non-heavy metal-exposed and non-menopausal), and group HN (heavy metal-exposed and non-menopausal).

Nodes are classified into four categories based on intra-module connectivity (*Zi*) and inter-module connectivity (*Pi*) metrics: peripherals (*Zi* ≤ 2.5, *Pi* ≤ 0.62), connectors (*Zi* ≤ 2.5, *Pi* ≥ 0.62), module hubs (*Zi* ≥ 2.5, *Pi* ≤ 0.62), and network hubs (*Zi* ≥ 2.5, *Pi* ≥ 0.62) (34, 35). The results showed that no nodes classified as network hubs were detected in any of the four groups of networks, and module hubs and connectors are generally regarded as keystone species that play a crucial role in maintaining network topology (36). Analysis results indicate (Fig. 10) that key species were detected in the NM, NN, and HN groups, while such key species were entirely absent in the HM group’s network.

## DISCUSSION

This study is the first to investigate the combined effects (or joint effects) of heavy metal exposure and menopausal status on the female oral microbiome. While previous research has separately detailed the impacts of each factor, how they synergistically influence (or jointly affect) the oral microbiome remains unclear. We propose and validate a core hypothesis: heavy metal exposure may disrupt host health by specifically altering the community structure, function, and ecological networks of oral microbes, potentially influencing the menopausal transition process in women. Through multidimensional analysis, this study found: (i) their combined effect (HM group) is most severe; (ii) Mo exhibits uniquely strong correlations with key genera; (iii) microbial protective functions are downregulated; and (iv) the synergistic effect precipitates a collapse in the microbial ecological network.

Both heavy metal exposure and menopausal status independently reduced oral microbial α-diversity, with the HM group showing the most pronounced impact. β-diversity analysis further revealed distinct community structures between exposed vs. unexposed groups and menopausal vs. non-menopausal groups (except NM and NN groups). These findings corroborate the role of heavy metals as an environmental selective pressure shaping microbial community structure (37, 38) and reflect the impact of physiological changes induced by menopause on oral microbiota (39).

At the phylum level, the heavy metal exposure groups (HM and HN) exhibited increased relative abundances of Actinobacteria and Proteobacteria, consistent with some previously observed patterns (26). However, the reduction in Firmicutes and Bacteroidetes alongside the trend in Proteobacteria observed in this study diverged from certain previous reports (40–44). This discrepancy may suggest that menopausal status, as a key host internal environmental factor, interacts with heavy metal exposure to jointly drive a unique state of microbial imbalance.

Building upon the community-wide shifts described above, our genus-level correlation analysis identified specific bacterial taxa whose abundances were intricately linked to heavy metal exposure and menopausal status, with Mo emerging as a pivotal mediator (Fig. 7B). For instance, the genus *Gemella* shows positive correlation with Mo while negatively correlating with most microbial metabolic pathways; the genus *Rothia* correlates positively with Ni, and its enrichment is accompanied by reduced abundance in pathways such as lipid and amino acid metabolism (37). Conversely, the genus *Rhodococcus*, significantly negatively correlated with Mo, exhibited decreased abundance associated with increased activity in multiple functional pathways. This indicates that heavy metal exposure enriches tolerant bacterial genera (e.g., *Gemella* and *Rothia*) while suppressing sensitive genera (e.g., *Rhodococcus* and *Leptotrichia*), thereby bidirectionally disrupting the functional equilibrium of the microbial community (26, 45).

The uniquely prominent role of Mo likely stems from its distinctive biochemical properties. In the oral environment, it exists as the highly bioavailable molybdate anion (MoO₄²⁻), which can be efficiently absorbed by bacteria through transport systems similar to those for sulfate/phosphate (46, 47). As an essential cofactor for key enzymes like nitrate reductase (48, 49), Mo likely promotes the growth of nitrate-reducing bacteria such as *Gemella* and *Rothia*, explaining the observed positive correlation. However, excessive molybdate competitively inhibits bacterial sulfate/phosphate metabolism, potentially constituting a toxic mechanism underlying reduced abundances of genera like *Rhodococcus* (50). Thus, Mo acts as a central mediator reshaping the functional landscape of the oral microbiota (Fig. 8) through dual effects of “nutrient supply” and “metabolic inhibition” (51).

This heavy metal-driven (particularly Mo) microbial dysregulation may be linked to systemic health risks in postmenopausal women. Disrupted oral microbial functions could influence stroke risk in elderly women by participating in metabolic pathways (52). More critically, such microbial imbalance may interfere with host endogenous hormonal homeostasis (38, 53). Multiple studies confirm significant associations between heavy metal exposure and reduced estrogen levels in postmenopausal women (54–58). This research indicates that alterations in oral microbial community structure and its unique functional metabolic profiles may represent a key mechanistic link through which heavy metal bioaccumulation impacts women’s health—particularly postmenopausal women—manifesting as oral issues, metabolic disorders, and cardiovascular risks.

The aforementioned alterations in bacterial genera and functions ultimately triggered a more profound structural collapse at the level of ecological association networks within the microbial community.

Co-occurrence network analysis in this study revealed that heavy metal exposure combined with menopausal status led to significant simplification of oral microbial network structure and loss of keystone species (HM group). In contrast, individuals exposed to heavy metals but not experiencing menopause (NN group) maintained a more complex and stable network. Keystone species play a pivotal role in microbial ecological networks, coordinating associations between different modules through high connectivity to maintain functional redundancy and stability within the community (59, 60). The complete absence of keystone species in the HM group network (Fig. 10) suggests that this network may have lost its core regulators and stabilizers. This directly explains why the HM group’s network topology features (e.g., low average degree and low graph density) exhibit structural “looseness” and “simplification” (Table 3). Networks lacking such key nodes are considered to have lower ecological resilience, making them more susceptible to structural collapse and community dysregulation when subjected to environmental disturbances (e.g., pathogen invasion and additional environmental stress) (61, 62).

The development of periodontitis is closely linked to dysbiosis in the oral microbiome. Studies reveal that healthy oral networks exhibit greater complexity and stability, whereas periodontal disease patients often display simplified, modularized microbial networks with loss of key symbiotic bacteria (63, 64). The network characteristics observed in the HM group in this study are similar, suggesting that the dual stressors of heavy metal exposure and menopause may create ecological conditions favorable for the proliferation of periodontal pathogens by disrupting network stability. Menopause is often accompanied by changes in saliva composition and flow rate (e.g., xerostomia). A fragile microbial network lacking keystone species may be less capable of adapting to such environmental shifts, thereby exacerbating dysbiosis and related clinical symptoms. Thus, the synergistic effects of heavy metal exposure and menopause not only alter species composition but also profoundly disrupt community topology, leading to keystone species loss and reduced network complexity. This network-level dysregulation likely represents a crucial mechanistic link connecting environmental exposure, physiological changes, and the heightened oral health risks observed in postmenopausal women.

The oral microbiome dysbiosis characterized in this study—marked by taxonomic shifts, functional decline, and network disintegration—may exert systemic effects on menopausal women’s health through the oral-systemic axis. We propose that these microbial alterations are not merely parallel outcomes of heavy metal exposure and menopause but are interconnected events that can initiate or exacerbate a pathophysiological cascade. This cascade likely involves the convergence of depleted microbial metabolic support, a consequent pro-inflammatory state facilitated by network instability, and the direct endocrine-disrupting effects of heavy metals. Together, they may form a vicious cycle that potentiates the metabolic and inflammatory burdens associated with the menopausal transition.

First, the downregulation of the microbiome’s cardiovascular and metabolic protective functions may weaken its positive regulatory role on host-related metabolic pathways. When this microbial support function is absent, combined with the inherent decline in estrogen levels during menopause (65, 66) and associated metabolic changes, it may collectively exacerbate conditions such as insulin resistance and lipid metabolism disorders. This partially explains the underlying mechanisms behind the increased risk of cardiovascular disease and diabetes in postmenopausal women (67–69).

Second, the collapse of ecological networks and loss of keystone species signifies severely impaired resilience against disturbances and compromised stability maintenance within microbial communities. This fragile ecological state readily induces or perpetuates chronic low-grade inflammation. Periodontitis, a prototypical chronic inflammatory oral disease, has been demonstrated to develop through similar microbial network simplification and dysbiosis. The network characteristics observed in the HM group suggest that heavy metal exposure and menopause may synergistically create an oral microenvironment conducive to chronic inflammation.

More importantly, the aforementioned microbe-driven local inflammation and metabolic dysregulation may converge with heavy metals' direct endocrine-disrupting effects along the same pathological pathway. Multiple lines of evidence indicate that heavy metal exposure can independently reduce estrogen (E2) levels in postmenopausal women. Therefore, we propose an integrative hypothesis: Heavy metal exposure induces profound dysregulation of the oral microbiome—manifesting as loss of protective functions, collapse of ecological networks, and associated chronic inflammation risks—which may synergize with the direct endocrine-disrupting effects of heavy metals. Together, these factors form a vicious cycle that accelerates or exacerbates menopausal metabolic disorders, immune dysregulation, and disruption of hormonal homeostasis.

This study has several important limitations. First, conclusions are based on a cross-sectional design, precluding causal inference, and a limited sample size may affect the robustness of some findings. Second, methodologically, 16S rRNA gene sequencing lacks strain-level resolution, and PICRUSt2-based functional prediction provides only preliminary inferences requiring validation through metagenomics and metabolomics. Furthermore, we did not collect detailed data on oral hygiene practices, which could influence the oral microbiome. Future studies should incorporate clinical oral health assessments to better control for this important factor. Most critically, this study did not directly measure serum hormone levels (e.g., estradiol, luteinizing hormone), preventing the establishment of a direct link between observed microbiome changes and host endocrine status. This limitation restricts direct confirmation of the core hypothesis that “the microbiome influences the menopausal process.” Future research should employ large-scale longitudinal designs to synchronously and dynamically monitor hormone profiles alongside multi-omics microbial data during the female menopausal transition. This approach will empirically elucidate the specific mechanistic pathways through which environmental exposures influence women’s health via the oral microbiome.

## MATERIALS AND METHODS

### Study site

Baiyin City in Gansu Province has serious heavy metal pollution in the environment of soil in the area due to long-term non-ferrous metal smelting activities in the last century. We selected Minqin village and Shuanghe village (36°28′ 38. 188 ″ N, 104°18′ 47. 870 ″ E; 36°27′ 24. 650 ″ N, 104°21′ 22. 057 ″ E) as typical polluted areas (H). For comparison, Hewan and Yangwa villages (35°46′ 41. 541 ″ N, 104°0′ 37. 443 ″ E; 35°45′ 54. 661 ″ N, 104°1′ 28. 117 ″ E), which are 100 km away from the area on the windward side, in Yuzhong County, Lanzhou City, and with a relatively low level of heavy metal pollution, were selected as non-polluted areas (N).

### Heavy metal analysis, human blood samples, and oral mucosa collection

All environmental samples collected were soil samples, not plant, vegetable, or fruit samples that participants might have consumed in their diet. Weigh 0.10 g of sieved soil into a digestion vessel. Add 6 mL HNO₃, 2 mL HF, and 2 mL H₂O₂. Perform programmed digestion using a microwave digestion system (180°C, hold for 20 min). After acid removal, dilute the digestion solution to 50 mL (using 2% HNO₃ as the diluent). Filter the solution prior to analysis. Collect venous blood using EDTA-K₂ anticoagulant vacuum tubes. Take 0.50 mL of whole blood, add 3 mL of HNO₃ and 1 mL of H₂O₂, and allow to stand overnight for pre-digestion. Digest the sample for 2 h on a 95°C hotplate. After cooling the digestion solution, dilute to a final volume of 10 mL with ultrapure water for subsequent analysis. Determined by ICP-MS (Agilent, USA) with online internal standard calibration (¹⁰³Rh, ¹⁸⁵Re), yielding standard curve correlation coefficients (r²) > 0.999. The method quantification limits (MQL) for each element ranged from 0.01 to 0.50 μg/L. Control was achieved through analysis of certified reference materials (soil: GBW07405; blood: Seronorm Trace Elements Whole Blood), yielding recovery rates of 85%–115% and within-batch precision (RSD) < 10%. The normal reference ranges for each blood heavy metal are listed in the supplementary materials (70, 71).

We used sterile cotton swabs to collect samples uniformly from the midpoint of the left buccal mucosa (the buccal area corresponding to the second molar) of each subject. After screening, a total of 47 samples were collected. All participant samples have undergone standardized processing. Apply moderate, even pressure to press the swab head firmly against the mucosa. Slowly roll the swab stick in the same direction across an area of approximately 2 × 2 cm, maintaining continuous rolling friction for about 30 s to ensure thorough contact. Repeat the rolling sampling process three times at the same site, using a different side of the swab each time. Immediately after sampling, immerse the swab head in a microcentrifuge tube pre-filled with 1.0–1.5 mL nucleic acid stabilizing solution. Place the collected sample tube into a portable dry ice container on-site immediately. Transport all samples to the laboratory within 4 h of collection. After verification, promptly transfer them to a −80°C ultra-low temperature freezer for long-term storage until downstream DNA/RNA extraction. This study was approved by the Ethics Committee of the School of Public Health, Lanzhou University (IRB18022701). Quality assurance/control procedures were performed for each batch of samples (one blank and one standard) using standard reference materials (National Institute of Metrology, Beijing, China).

### Definition of menopausal status

According to the recommendations of the World Health Organization (72) and the internationally recognized STRAW+10 staging system (73), this study defines natural menopause as: age ≥40 years, spontaneous amenorrhea for ≥12 months, and exclusion of other pathological or iatrogenic factors. All participants in this study were naturally menopausal.

### Study population

From September 2019 to September 2021, we recruited women aged from 40 to 60 years in heavy metal-polluted and non-polluted areas who were willing to participate in and cooperate with the study. According to the menopausal status at that time, menopausal women were categorized as the menopausal group (Group M), and those who were not yet menopausal were categorized as the non-menopausal group (Group N). All participants met the following criteria: (i) female, signed informed consent prior to sampling, and had not used any antibiotics for at least 2 weeks; (ii) had lived in the area for more than 10 years; and (iii) had a stable local residence and had not worked outside the home for more than 6 months consecutively. Exclusion criteria were as follows: (i) presence of immune system disorders and (ii) a history of severe, progressive, or uncontrolled cardiac, hepatic, renal, psychiatric, or hematologic disorders. Based on the above requirements, a total of 47 female participants were finally enrolled in the study, which were then categorized into four groups based on whether they came from heavy metal-contaminated areas: group NM (non-heavy metal-exposed and post-menopausal, *n* = 11), group HM (heavy metal-exposed and post-menopausal, *n* = 18), group NN (non-heavy metal-exposed and non-menopausal, *n* = 5), and group HN (heavy metal-exposed and non-menopausal, *n* = 13).

### DNA extraction, sequencing, and bioinformatics analysis

The total genomic DNA of the buccal mucosa samples was extracted with the E.Z.N.A. Soil DNA Kit (OMEGA, USA) according to the instructions. The quality of the extracted genomic DNA was measured by agarose gel electrophoresis with 1% agarose, and the concentration and purity of the DNA were determined by using the NanoDrop2000 (Thermo). The extracted genomic DNA of buccal mucosa was amplified in the V3–V4 region with the upstream primer ACTCCTACGGGGAGGCAGCAG and the downstream primer GGACTACHVGGGGTWTCTAAT by an ABI GeneAmp 9700 PCR instrument (ABI, CA, USA). Initial denaturation at 95°C for 3 min, followed by 27 cycles of denaturing at 95°C for 30 s, annealing at 55°C for 30 s, and extension at 72°C for 30 s, and single extension at 72°C for 10 min, and end at 4°C. All amplification reactions were performed in a total volume of 20 μL containing 4 μL of 5× FastPfu buffer, 2 μL of 2.5 mM dNTP, 0.8 μL of forward and reverse primers, 10 ng of template DNA, and 0.4 μL of FastPfu DNA polymerase. Three replicates were performed for each sample. PCR products from the same sample were mixed and PCR products were recovered using a 2% agarose gel, and the recovered products were purified using the AxyPrep DNA Gel Extraction Kit (Axygen Biosciences, Union City, CA, USA), detected by electrophoresis on a 2% agarose gel and analyzed using a Quantus Fluorometer (Promega, USA) was used to quantify the recovered products. Purified amplicons were combined at equimolar concentrations and paired-end sequenced (2 × 300  bp) on an Illumina MiSeq platform (Illumina, United States) at the Majorbio Bio-pharm Technology Co., Ltd. (Shanghai, China) according to standard protocols. Raw sequencing data of the bacterial 16S rRNA gene has been deposited in the NCBI Sequence Read Archive under BioProject accession number PRJNA979792. This BioProject contains sequence data from multiple related studies. The 47 buccal mucosa samples analyzed in the present study correspond to the SRA Sample Accessions SAMN35578740 through SAMN35578870. The detailed correspondence between each sample’s accession (SAMN), its original identifier, and its assigned experimental group (NM, HM, NN, or HN) is provided in Table S10. The resulting sequences were processed using the QIIME pipeline. Briefly, low-quality sequences were trimmed with Cutadapt and quality filtered. Paired-end reads were assembled using FLASH version 1.2.11. USEARCH was used to remove chimeric sequences based on the UCHIME algorithm, and the remaining sequences were allocated to operational taxonomic units (OTUs) with 97% similarity using the UPARSE pipeline. OTUs with fewer than two sequences were eliminated, and their representative sequences were assigned to taxonomic lineages using the RDP classifier version 2.2 against the SILVA database (version 138) using a confidence threshold of 0.7.

### Statistical analysis

Alpha diversity indices such as Shannon Index, Richness, Chao1, and Simpson Index were calculated using Qiime software. The Wilcoxon rank-sum test was employed for intergroup differences in alpha diversity. The similarity of microbial community structure among samples was examined using PCA analysis (Principal Component Analysis), and statistical significance was assessed by PERMANOVA non-parametric test. The Wilcoxon rank-sum test was used to compare the dominant bacteria at the phylum and genus level (phyla and genera with relative abundance greater than 1% were considered to be the dominant taxa), as well as the relative abundance of taxa at the level of selected species, in the exposed and control groups. PICRUSt2 was used to analyze the 16S rRNA gene-based prediction of metabolic function in human oral bacteria. Spearman’s rank correlation was calculated between pairs of OTUs, and *P*-values from the correlation analysis were adjusted using the Benjamini and Hochberg False Discovery Rate (FDR) controlling methods. Significance was defined as q-value < 0.05. The meta-community network was constructed using an online tool called the Molecular Ecosystem Network Analysis Pipeline (MENAP), based on the correlation coefficients and FDR-adjusted *P*-values. A cutoff of 0.001 for *P*-values (FDR-adjusted) and a threshold of 0.77 for correlation coefficients were selected using the methods dependent on random matrix theory. Network topological features were obtained with the “igraph” package. Network images were visualized using Gephi 0.10.1 (74) (https://gephi.org/). Potential confounding factors (smoking, alcohol consumption, and meal structure) were compared across groups (Table 1). As no significant differences were detected (*P* > 0.05), and given the sample size constraints, these variables were not included as covariates in the primary microbial community analyses to preserve statistical power and avoid model overfitting.

## Data Availability

The data sets presented in this study can be found in online repositories. The names of the repository/repositories and accession number(s) can be found below: NCBI BioProject PRJNA979792.

## SUPPLEMENTAL MATERIAL

Supplemental tables spectrum.03123-25-s0001.docx Tables S1 to S10.

ASM does not own the copyrights to Supplemental Material that may be linked to, or accessed through, an article. The authors have granted ASM a non-exclusive, world-wide license to publish the Supplemental Material files. Please contact the corresponding author directly for reuse.
