# Supplementary material for: Effects of heavy metal exposure on oral microbial communities in women with different menopausal status
Source: Microbiol Spectr. 2026 Mar 23;14(5):e03123-25. doi: 10.1128/spectrum.03123-25 (PMC13142029; doi:10.1128/spectrum.03123-25)
Supplement: Supplemental tables — Tables S1 to S10. [file spectrum.03123-25-s0001.docx]

**Supplementary Material**

**Table S1.** The concentration of metals in soils between contaminated and control areas (unit: mg/kg).

| Element | H (n = 15) | N (n = 13) | *P*-value |
| --- | --- | --- | --- |
| Co | 4.75 ± 0.14 | 7.36 ± 0.26 | <0.001 |
| Mn | 316.25 ± 10.24 | 422.11 ± 13.03 | <0.001 |
| Pb | 100.77 ± 13.97 | 13.40 ± 0.30 | <0.001 |
| Cd | 6.03 ± 1.01 | 0.17 ± 0.01 | <0.001 |
| Sb | 1.83 ± 0.18 | 0.93 ± 0.02 | <0.001 |
| Zn | 294.38 ± 47.93 | 52.21 ± 1.69 | <0.001 |
| Cu | 50.00 ± 6.25 | 29.27 ± 4.24 | 0.011 |
| Hg | 0.45 ± 0.14 | 0.02 ± 0.01 | 0.026 |
| Mo | 0.53 ± 0.03 | 0.60 ± 0.02 | 0.112 |
| Ni | 258.18 ± 46.31 | 169.30 ± 33.11 | 0.136 |

Note: H, contaminated areas; N, control areas. Data were shown as mean ± SE.

**Table S2.** The concentration of metals in the blood of subjects living in both contaminated and control areas (unit: μg/L).

| Element | H (n = 23) | N (n = 4) | Reference Range(μg/L) | *P*-value |
| --- | --- | --- | --- | --- |
| Cd | 8.19 ± 0.97 | 0.85 ± 0.11 | 0.2-0.5 | <0.001 |
| Pb | 49.60 ± 4.45 | 15.88 ± 1.36 | 10-20 | <0.001 |
| Zn | 5765.61 ± 206.52 | 4684.60 ± 434.67 | 4000-7000 | 0.270 |
| Cu | 861.36 ± 28.07 | 759.33 ± 50.25 | 700-1100 | 0.338 |
| Ni | 3.52 ± 0.79 | 6.54 ± 2.09 | 0.1-0.5 | 0.501 |
| Mn | 20.27 ± 2.67 | 16.61 ± 2.88 | 8-12 | 0.554 |
| Mo | 2.82 ± 0.53 | 2.20 ± 0.45 | 0.5-3.0 | 0.554 |
| Co | 0.47 ± 0.07 | 0.41 ± 0.04 | 0.1-0.5 | 0.555 |
| Hg | 0.58 ± 0.05 | 0.51 ± 0.11 | 0.5-2.0 | 0.555 |
| Sb | 0.10 ± 0.02 | 0.13 ± 0.04 | 0.1-0.3 | 0.555 |

Note: H, contaminated areas; N, control areas. Data were shown as mean ± SE.

**Table S3.** Significant secondary KEGG functional pathway results for the NM group and HM group.

| Pathway | log2(FC) | Regulation |
| --- | --- | --- |
| Endocrine system | -0.284982994 | Sig_Down |
| Transport and catabolism | -0.306994346 | Sig_Down |
| Metabolism of terpenoids and polyketides | -0.356956943 | Sig_Down |
| Cancer: specific types | -0.558280022 | Sig_Down |
| Cardiovascular disease | -0.400760805 | Sig_Down |
| Substance dependence | -0.769460823 | Sig_Down |
| Lipid metabolism | -0.318737473 | Sig_Down |
| Nervous system | -0.30779209 | Sig_Down |
| Xenobiotics biodegradation and metabolism | -0.437074552 | Sig_Down |
| Environmental adaptation | -0.321293836 | Sig_Down |
| Neurodegenerative disease | -0.316022381 | Sig_Down |
| Excretory system | -0.507181202 | Sig_Down |
| Infectious disease: parasitic | -0.532652857 | Sig_Down |
| Chemical structure transformation maps | -0.491906834 | Sig_Down |
| Cell motility | -0.334165577 | Sig_Down |

**Table S4.** Significant secondary KEGG functional pathway results for the NN group and HN group.

| Pathway | log2(FC) | Regulation |
| --- | --- | --- |
| Cellular community - eukaryotes | 2.825408106 | Sig_Up |
| Signaling molecules and interaction | 2.830283282 | Sig_Up |
| Cell growth and death | -0.44380038 | Sig_Down |
| Cellular community - prokaryotes | -0.407218283 | Sig_Down |
| Transport and catabolism | -1.322332573 | Sig_Down |
| Signal transduction | -0.507052798 | Sig_Down |
| Folding, sorting and degradation | -0.371866525 | Sig_Down |
| Cancer: overview | -0.738476634 | Sig_Down |
| Cancer: specific types | -1.446187266 | Sig_Down |
| Endocrine and metabolic disease | -0.359601067 | Sig_Down |
| Immune disease | -0.588828679 | Sig_Down |
| Neurodegenerative disease | -1.189833315 | Sig_Down |
| Substance dependence | -5.555680883 | Sig_Down |
| Amino acid metabolism | -0.963891668 | Sig_Down |
| Biosynthesis of other secondary metabolites | -0.702468428 | Sig_Down |
| Carbohydrate metabolism | -0.635994981 | Sig_Down |
| Chemical structure transformation maps | -2.514081921 | Sig_Down |
| Energy metabolism | -0.580999849 | Sig_Down |
| Global and overview maps | -0.746697073 | Sig_Down |
| Lipid metabolism | -1.608834843 | Sig_Down |
| Metabolism of cofactors and vitamins | -0.458383283 | Sig_Down |
| Metabolism of other amino acids | -0.439206251 | Sig_Down |
| Metabolism of terpenoids and polyketides | -1.711894197 | Sig_Down |
| Xenobiotics biodegradation and metabolism | -2.398150129 | Sig_Down |
| Aging | -0.799044125 | Sig_Down |
| Endocrine system | -1.090318496 | Sig_Down |
| Environmental adaptation | -1.14678878 | Sig_Down |
| Excretory system | -1.388497849 | Sig_Down |
| Immune system | -0.880003788 | Sig_Down |
| Nervous system | -1.148210176 | Sig_Down |
| Infectious disease: parasitic | -1.24228701 | Sig_Down |
| Cardiovascular disease | -0.751409271 | Sig_Down |
| Cell motility | -1.118746465 | Sig_Down |
| Infectious disease: viral | -0.448555649 | Sig_Down |

**Table S5.** Significant secondary KEGG functional pathway results for the HM group and HN group.

| Pathway | log2(FC) | Regulation |
| --- | --- | --- |
| Substance dependence | -1.129639233 | Sig_Down |
| Immune system | -0.556613947 | Sig_Down |
| Nervous system | -0.549034424 | Sig_Down |
| Metabolism of terpenoids and polyketides | -0.70308496 | Sig_Down |
| Environmental adaptation | -0.581070472 | Sig_Down |
| Lipid metabolism | -0.662093313 | Sig_Down |
| Transport and catabolism | -0.631471864 | Sig_Down |
| Xenobiotics biodegradation and metabolism | -0.830794846 | Sig_Down |
| Endocrine system | -0.500348312 | Sig_Down |
| Amino acid metabolism | -0.442115282 | Sig_Down |
| Global and overview maps | -0.352864988 | Sig_Down |
| Carbohydrate metabolism | -0.312135801 | Sig_Down |
| Energy metabolism | -0.29296464 | Sig_Down |
| Aging | -0.387983391 | Sig_Down |
| Cancer: overview | -0.351192427 | Sig_Down |
| Excretory system | -0.630812456 | Sig_Down |
| Infectious disease: parasitic | -0.655047259 | Sig_Down |
| Cell motility | -0.763979029 | Sig_Down |
| Cancer: specific types | -0.646607847 | Sig_Down |
| Biosynthesis of other secondary metabolites | -0.348193357 | Sig_Down |
| Chemical structure transformation maps | -0.858652072 | Sig_Down |
| Cardiovascular disease | -0.389984421 | Sig_Down |
| Neurodegenerative disease | -0.544175037 | Sig_Down |
| Infectious disease: viral | -0.369272081 | Sig_Down |

**Table S6.** Significant results for the tertiary KEGG functional pathways between the NM group and HM group.

| Pathway | log2(FC) | Regulation |
| --- | --- | --- |
| Lysosome | 0.603422947 | Sig_Up |
| N-Glycan biosynthesis | 0.314948477 | Sig_Up |
| RIG-I-like receptor signaling pathway | 0.534695757 | Sig_Up |
| Biosynthesis of ansamycins | 0.315905537 | Sig_Up |
| Isoflavonoid biosynthesis | 1.965307284 | Sig_Up |
| Other glycan degradation | 0.44955094 | Sig_Up |
| Staphylococcus aureus infection | 0.365803329 | Sig_Up |
| Carbohydrate digestion and absorption | 0.334478685 | Sig_Up |
| Apoptosis - fly | -0.383065852 | Sig_Down |
| Basal transcription factors | -0.875023301 | Sig_Down |
| Prion diseases | -0.719654225 | Sig_Down |
| Atrazine degradation | -0.734148895 | Sig_Down |
| Glyoxylate and dicarboxylate metabolism | -0.3522643 | Sig_Down |
| Sulfur metabolism | -0.359911224 | Sig_Down |
| Lipoarabinomannan (LAM) biosynthesis | -0.80856275 | Sig_Down |
| Longevity regulating pathway - multiple species | -0.312946113 | Sig_Down |
| PPAR signaling pathway | -0.513420456 | Sig_Down |
| Peroxisome | -0.409433075 | Sig_Down |
| Phenylalanine metabolism | -0.607539088 | Sig_Down |
| Tyrosine metabolism | -0.470533665 | Sig_Down |
| Propanoate metabolism | -0.288124218 | Sig_Down |
| Fatty acid metabolism | -0.276900222 | Sig_Down |
| MAPK signaling pathway - yeast | -0.493661999 | Sig_Down |
| Proteasome | -0.718411446 | Sig_Down |
| Arginine biosynthesis | -0.457613932 | Sig_Down |
| Histidine metabolism | -0.667281399 | Sig_Down |
| Tryptophan metabolism | -0.623182606 | Sig_Down |
| Valine, leucine and isoleucine degradation | -0.485325497 | Sig_Down |
| Caffeine metabolism | -0.774713755 | Sig_Down |
| Butanoate metabolism | -0.386186064 | Sig_Down |
| Inositol phosphate metabolism | -0.375089391 | Sig_Down |
| Biosynthesis of unsaturated fatty acids | -0.372829188 | Sig_Down |
| Fatty acid degradation | -0.563917978 | Sig_Down |
| Phosphonate and phosphinate metabolism | -0.361661317 | Sig_Down |
| Caprolactam degradation | -0.721330181 | Sig_Down |
| Thyroid hormone signaling pathway | -0.75228087 | Sig_Down |
| Meiosis - yeast | -0.641837951 | Sig_Down |
| Fluid shear stress and atherosclerosis | -0.398562798 | Sig_Down |
| Amyotrophic lateral sclerosis (ALS) | -0.538171101 | Sig_Down |
| Alcoholism | -0.769460823 | Sig_Down |
| Amphetamine addiction | -0.769460823 | Sig_Down |
| Cocaine addiction | -0.769460823 | Sig_Down |
| Citrate cycle (TCA cycle) | -0.316979178 | Sig_Down |
| Steroid biosynthesis | -0.768940996 | Sig_Down |
| Steroid hormone biosynthesis | -0.76074916 | Sig_Down |
| alpha-Linolenic acid metabolism | -0.630646024 | Sig_Down |
| beta-Alanine metabolism | -0.528339621 | Sig_Down |
| Carotenoid biosynthesis | -0.626507587 | Sig_Down |
| Geraniol degradation | -0.676098671 | Sig_Down |
| Limonene and pinene degradation | -0.657480207 | Sig_Down |
| Renin-angiotensin system | -0.568270284 | Sig_Down |
| Dopaminergic synapse | -0.769626645 | Sig_Down |
| Serotonergic synapse | -0.769301075 | Sig_Down |
| Ferroptosis | -0.310170657 | Sig_Down |
| Non-homologous end-joining | -0.656123244 | Sig_Down |
| Pathways in cancer | -0.510097905 | Sig_Down |
| Novobiocin biosynthesis | -0.329497986 | Sig_Down |
| Nitrogen metabolism | -0.328616655 | Sig_Down |
| Synthesis and degradation of ketone bodies | -0.47831584 | Sig_Down |
| Ethylbenzene degradation | -0.665249344 | Sig_Down |
| Fluorobenzoate degradation | -0.682356566 | Sig_Down |
| Nitrotoluene degradation | -0.589589323 | Sig_Down |
| Styrene degradation | -0.444695111 | Sig_Down |
| Toluene degradation | -0.686849346 | Sig_Down |
| Insulin signaling pathway | -0.319169853 | Sig_Down |
| GABAergic synapse | -0.298039652 | Sig_Down |
| Bladder cancer | -0.813551116 | Sig_Down |
| Chemical carcinogenesis | -0.540713533 | Sig_Down |
| Insect hormone biosynthesis | -0.451432706 | Sig_Down |
| Nonribosomal peptide structures | -0.762103701 | Sig_Down |
| Aminobenzoate degradation | -0.442692686 | Sig_Down |
| Benzoate degradation | -0.506884187 | Sig_Down |
| Drug metabolism - cytochrome P450 | -0.46130084 | Sig_Down |
| Metabolism of xenobiotics by cytochrome P450 | -0.448782973 | Sig_Down |
| Adipocytokine signaling pathway | -0.587853051 | Sig_Down |
| Glutamatergic synapse | -0.269437357 | Sig_Down |
| cAMP signaling pathway | -0.773478419 | Sig_Down |
| Steroid degradation | -0.775976939 | Sig_Down |
| Hematopoietic cell lineage | -0.775996241 | Sig_Down |
| PI3K-Akt signaling pathway | -0.568586734 | Sig_Down |
| Protein processing in endoplasmic reticulum | -0.621858905 | Sig_Down |
| Hepatocellular carcinoma | -0.478525449 | Sig_Down |
| Lysine degradation | -0.431722645 | Sig_Down |
| Degradation of aromatic compounds | -0.402186035 | Sig_Down |
| Retinol metabolism | -0.481491277 | Sig_Down |
| Thermogenesis | -0.552876312 | Sig_Down |
| Primary bile acid biosynthesis | -0.710195495 | Sig_Down |
| Chloroalkane and chloroalkene degradation | -0.345597471 | Sig_Down |
| Oxidative phosphorylation | -0.278403207 | Sig_Down |
| Naphthalene degradation | -0.297919387 | Sig_Down |
| Polycyclic aromatic hydrocarbon degradation | -0.545015126 | Sig_Down |
| NOD-like receptor signaling pathway | -0.263922054 | Sig_Down |
| Linoleic acid metabolism | -0.591004227 | Sig_Down |
| Ubiquinone and other terpenoid-quinone biosynthesis | -0.449883562 | Sig_Down |
| Mannose type O-glycan biosynthesis | -1.353636955 | Sig_Down |
| Other types of O-glycan biosynthesis | -1.353636955 | Sig_Down |
| Streptomycin biosynthesis | -0.322804558 | Sig_Down |
| Tropane, piperidine and pyridine alkaloid biosynthesis | -0.283252261 | Sig_Down |
| Porphyrin and chlorophyll metabolism | -0.414815548 | Sig_Down |
| Dioxin degradation | -0.298735163 | Sig_Down |
| Proximal tubule bicarbonate reclamation | -0.507176465 | Sig_Down |
| Phenylpropanoid biosynthesis | -0.508198618 | Sig_Down |
| African trypanosomiasis | -0.585395855 | Sig_Down |
| Chagas disease (American trypanosomiasis) | -0.587024121 | Sig_Down |
| Acarbose and validamycin biosynthesis | -0.608148886 | Sig_Down |
| Ether lipid metabolism | -0.516838593 | Sig_Down |
| Prostate cancer | -0.539756997 | Sig_Down |
| Renal cell carcinoma | -0.598260916 | Sig_Down |
| Cushing syndrome | -0.598260916 | Sig_Down |
| Polyketide sugar unit biosynthesis | -0.606776956 | Sig_Down |
| Estrogen signaling pathway | -0.539756998 | Sig_Down |
| Progesterone-mediated oocyte maturation | -0.539756998 | Sig_Down |
| Antigen processing and presentation | -0.539756998 | Sig_Down |
| IL-17 signaling pathway | -0.539755724 | Sig_Down |
| Th17 cell differentiation | -0.539756998 | Sig_Down |
| Bacterial chemotaxis | -0.308010217 | Sig_Down |
| Biosynthesis of terpenoids and steroids | -0.491906834 | Sig_Down |
| Autophagy - yeast | -0.560101431 | Sig_Down |
| Salmonella infection | -0.374906066 | Sig_Down |
| Secondary bile acid biosynthesis | -0.578796111 | Sig_Down |
| Biosynthesis of vancomycin group antibiotics | -0.635544083 | Sig_Down |
| Apoptosis - multiple species | -0.33378443 | Sig_Down |
| p53 signaling pathway | -0.33378443 | Sig_Down |
| Colorectal cancer | -0.33378443 | Sig_Down |
| Small cell lung cancer | -0.333814091 | Sig_Down |
| Viral myocarditis | -0.33378443 | Sig_Down |
| Toxoplasmosis | -0.333558685 | Sig_Down |
| Hepatitis B | -0.333369466 | Sig_Down |
| Herpes simplex virus 1 infection | -0.332954642 | Sig_Down |
| Human cytomegalovirus infection | -0.333814091 | Sig_Down |
| Influenza A | -0.33328974 | Sig_Down |
| Kaposi sarcoma-associated herpesvirus infection | -0.333814092 | Sig_Down |
| Riboflavin metabolism | -0.286230804 | Sig_Down |
| Biosynthesis of siderophore group nonribosomal peptides | -0.466972659 | Sig_Down |
| Flagellar assembly | -0.391813357 | Sig_Down |
| Isoquinoline alkaloid biosynthesis | -0.424293938 | Sig_Down |
| Biofilm formation - Pseudomonas aeruginosa | -0.284899979 | Sig_Down |
| Sphingolipid signaling pathway | -0.419834696 | Sig_Down |
| Betalain biosynthesis | -0.464296688 | Sig_Down |
| Choline metabolism in cancer | -0.5956257 | Sig_Down |
| Glycosaminoglycan degradation | -0.292818684 | Sig_Down |
| Flavonoid biosynthesis | -0.987006392 | Sig_Down |
| Stilbenoid, diarylheptanoid and gingerol biosynthesis | -0.987006392 | Sig_Down |
| Phospholipase D signaling pathway | -0.578716974 | Sig_Down |
| Mineral absorption | -0.323601684 | Sig_Down |

**Table S7.** Significant results for the tertiary KEGG functional pathways between the NN group and HN group.

| Pathway | log2(FC) | Regulation |
| --- | --- | --- |
| Other glycan degradation | 0.829735996 | Sig_Up |
| Carbohydrate digestion and absorption | 0.748607029 | Sig_Up |
| NF-kappa B signaling pathway | 4.378511623 | Sig_Up |
| TNF signaling pathway | 4.378511623 | Sig_Up |
| VEGF signaling pathway | 4.378511602 | Sig_Up |
| Leishmaniasis | 4.378511655 | Sig_Up |
| Ovarian steroidogenesis | 4.378511602 | Sig_Up |
| C-type lectin receptor signaling pathway | 4.548436625 | Sig_Up |
| Glycosphingolipid biosynthesis - ganglio series | 1.216629906 | Sig_Up |
| Various types of N-glycan biosynthesis | 1.217151458 | Sig_Up |
| Salivary secretion | 3.963474116 | Sig_Up |
| Biosynthesis of type II polyketide products | 4.741081691 | Sig_Up |
| Type I polyketide structures | 12.08607852 | Sig_Up |
| Fatty acid elongation | 4.317111079 | Sig_Up |
| D-Alanine metabolism | 0.279398584 | Sig_Up |
| Biosynthesis of ansamycins | 0.344743529 | Sig_Up |
| Oxytocin signaling pathway | 3.115477217 | Sig_Up |
| Lysosome | 0.691900653 | Sig_Up |
| RNA transport | 0.529230189 | Sig_Up |
| Furfural degradation | 2.702253289 | Sig_Up |
| Indole alkaloid biosynthesis | 3.108904561 | Sig_Up |
| Isoflavonoid biosynthesis | 4.862327403 | Sig_Up |
| Adherens junction | 10.9657843 | Sig_Up |
| Chemokine signaling pathway | 9.596550497 | Sig_Up |
| Fc gamma R-mediated phagocytosis | 11.01469387 | Sig_Up |
| Staphylococcus aureus infection | 0.772978274 | Sig_Up |
| Staurosporine biosynthesis | 2.429993528 | Sig_Up |
| Retrograde endocannabinoid signaling | 2.925999429 | Sig_Up |
| cGMP-PKG signaling pathway | 3.185866581 | Sig_Up |
| Adrenergic signaling in cardiomyocytes | 3.185866564 | Sig_Up |
| Gastric acid secretion | 3.18586652 | Sig_Up |
| Aldosterone synthesis and secretion | 3.185866532 | Sig_Up |
| Phosphotransferase system (PTS) | 0.339306775 | Sig_Up |
| Flavone and flavonol biosynthesis | 2.054585797 | Sig_Up |
| Tight junction | 3.548436625 | Sig_Up |
| Biosynthesis of enediyne antibiotics | 4.837943261 | Sig_Up |
| Endocrine and other factor-regulated calcium reabsorption | 2.600904047 | Sig_Up |
| Dilated cardiomyopathy (DCM) | 2.829230575 | Sig_Up |
| Hypertrophic cardiomyopathy (HCM) | 2.793266767 | Sig_Up |
| Glycosphingolipid biosynthesis - globo and isoglobo series | 0.713046686 | Sig_Up |
| Bisphenol degradation | 2.150242636 | Sig_Up |
| Regulation of actin cytoskeleton | 2.831273549 | Sig_Up |
| Focal adhesion | 2.829979072 | Sig_Up |
| Cell adhesion molecules (CAMs) | 2.831375983 | Sig_Up |
| ECM-receptor interaction | 2.830194898 | Sig_Up |
| Arrhythmogenic right ventricular cardiomyopathy (ARVC) | 2.829979071 | Sig_Up |
| Gap junction | 9.405069369 | Sig_Up |
| Biosynthesis of 12-, 14- and 16-membered macrolides | 11.9251423 | Sig_Up |
| Aldosterone-regulated sodium reabsorption | 2.963474118 | Sig_Up |
| Parathyroid hormone synthesis, secretion and action | 2.493988831 | Sig_Up |
| Malaria | 1.9634741 | Sig_Up |
| Apoptosis - fly | -0.929104441 | Sig_Down |
| Ferroptosis | -1.444630935 | Sig_Down |
| Meiosis - yeast | -3.299170842 | Sig_Down |
| Necroptosis | -0.541313349 | Sig_Down |
| Quorum sensing | -0.510564158 | Sig_Down |
| Peroxisome | -1.580657528 | Sig_Down |
| ABC transporters | -0.481897252 | Sig_Down |
| FoxO signaling pathway | -0.901039327 | Sig_Down |
| HIF-1 signaling pathway | -0.358921678 | Sig_Down |
| MAPK signaling pathway - plant | -0.45520094 | Sig_Down |
| MAPK signaling pathway - yeast | -1.929432801 | Sig_Down |
| Sphingolipid signaling pathway | -3.201365155 | Sig_Down |
| Two-component system | -0.561675305 | Sig_Down |
| cAMP signaling pathway | -7.828826182 | Sig_Down |
| Proteasome | -2.791374217 | Sig_Down |
| Protein export | -0.28278961 | Sig_Down |
| Protein processing in endoplasmic reticulum | -2.130530885 | Sig_Down |
| Sulfur relay system | -0.581009714 | Sig_Down |
| Non-homologous end-joining | -5.024453043 | Sig_Down |
| Basal transcription factors | -3.335441659 | Sig_Down |
| Ribosome biogenesis in eukaryotes | -0.329664127 | Sig_Down |
| Central carbon metabolism in cancer | -0.354830555 | Sig_Down |
| Chemical carcinogenesis | -2.863753292 | Sig_Down |
| MicroRNAs in cancer | -0.793383752 | Sig_Down |
| Pathways in cancer | -1.305574188 | Sig_Down |
| Bladder cancer | -3.008189213 | Sig_Down |
| Hepatocellular carcinoma | -1.498135263 | Sig_Down |
| Fluid shear stress and atherosclerosis | -0.824207918 | Sig_Down |
| Insulin resistance | -0.339561769 | Sig_Down |
| Type I diabetes mellitus | -0.50430719 | Sig_Down |
| Primary immunodeficiency | -0.601405053 | Sig_Down |
| Legionellosis | -0.523871389 | Sig_Down |
| Tuberculosis | -0.472221982 | Sig_Down |
| Amyotrophic lateral sclerosis (ALS) | -1.892913025 | Sig_Down |
| Prion diseases | -2.231609892 | Sig_Down |
| Alcoholism | -5.554906167 | Sig_Down |
| Amphetamine addiction | -5.554906167 | Sig_Down |
| Cocaine addiction | -5.556686173 | Sig_Down |
| Alanine, aspartate and glutamate metabolism | -0.642588541 | Sig_Down |
| Arginine and proline metabolism | -1.132201278 | Sig_Down |
| Arginine biosynthesis | -1.228111678 | Sig_Down |
| Cysteine and methionine metabolism | -0.314638311 | Sig_Down |
| Glycine, serine and threonine metabolism | -0.582968998 | Sig_Down |
| Histidine metabolism | -1.398803977 | Sig_Down |
| Lysine degradation | -2.265823656 | Sig_Down |
| Phenylalanine metabolism | -3.087415132 | Sig_Down |
| Phenylalanine, tyrosine and tryptophan biosynthesis | -0.26879278 | Sig_Down |
| Tryptophan metabolism | -3.611689178 | Sig_Down |
| Tyrosine metabolism | -2.072486897 | Sig_Down |
| Valine, leucine and isoleucine biosynthesis | -0.511861419 | Sig_Down |
| Valine, leucine and isoleucine degradation | -2.502502356 | Sig_Down |
| Betalain biosynthesis | -1.765875751 | Sig_Down |
| Caffeine metabolism | -8.76011327 | Sig_Down |
| Monobactam biosynthesis | -0.779437275 | Sig_Down |
| Neomycin, kanamycin and gentamicin biosynthesis | -0.732611151 | Sig_Down |
| Novobiocin biosynthesis | -0.848289505 | Sig_Down |
| Penicillin and cephalosporin biosynthesis | -0.950258299 | Sig_Down |
| Phenylpropanoid biosynthesis | -1.96943051 | Sig_Down |
| Prodigiosin biosynthesis | -0.848687378 | Sig_Down |
| Streptomycin biosynthesis | -0.793506842 | Sig_Down |
| Ascorbate and aldarate metabolism | -1.333454556 | Sig_Down |
| Butanoate metabolism | -1.633404423 | Sig_Down |
| Citrate cycle (TCA cycle) | -0.934242752 | Sig_Down |
| Glycolysis / Gluconeogenesis | -0.523447873 | Sig_Down |
| Glyoxylate and dicarboxylate metabolism | -1.391066628 | Sig_Down |
| Inositol phosphate metabolism | -1.588858647 | Sig_Down |
| Pentose and glucuronate interconversions | -0.689794581 | Sig_Down |
| Propanoate metabolism | -1.312402656 | Sig_Down |
| Pyruvate metabolism | -0.689951156 | Sig_Down |
| Biosynthesis of terpenoids and steroids | -2.514081921 | Sig_Down |
| Carbon fixation pathways in prokaryotes | -0.697041005 | Sig_Down |
| Methane metabolism | -0.682067993 | Sig_Down |
| Nitrogen metabolism | -1.155222032 | Sig_Down |
| Oxidative phosphorylation | -0.740206613 | Sig_Down |
| Sulfur metabolism | -1.517320809 | Sig_Down |
| 2-Oxocarboxylic acid metabolism | -0.598984488 | Sig_Down |
| Biosynthesis of amino acids | -0.36395994 | Sig_Down |
| Biosynthesis of secondary metabolites | -0.541656911 | Sig_Down |
| Carbon metabolism | -0.674272333 | Sig_Down |
| Degradation of aromatic compounds | -2.466943815 | Sig_Down |
| Fatty acid metabolism | -1.316522652 | Sig_Down |
| Metabolic pathways | -0.544269007 | Sig_Down |
| Microbial metabolism in diverse environments | -1.104374022 | Sig_Down |
| Lipoarabinomannan (LAM) biosynthesis | -3.29225338 | Sig_Down |
| Biosynthesis of unsaturated fatty acids | -1.747729627 | Sig_Down |
| Ether lipid metabolism | -3.352762271 | Sig_Down |
| Fatty acid biosynthesis | -0.543521603 | Sig_Down |
| Fatty acid degradation | -2.908517861 | Sig_Down |
| Glycerolipid metabolism | -0.583738881 | Sig_Down |
| Glycerophospholipid metabolism | -0.488139744 | Sig_Down |
| Linoleic acid metabolism | -4.154529887 | Sig_Down |
| Primary bile acid biosynthesis | -4.581669124 | Sig_Down |
| Secondary bile acid biosynthesis | -2.697257949 | Sig_Down |
| Steroid biosynthesis | -7.975855315 | Sig_Down |
| Steroid hormone biosynthesis | -5.886548317 | Sig_Down |
| Synthesis and degradation of ketone bodies | -2.773474782 | Sig_Down |
| alpha-Linolenic acid metabolism | -4.588039469 | Sig_Down |
| Biotin metabolism | -0.619363104 | Sig_Down |
| Lipoic acid metabolism | -0.419179628 | Sig_Down |
| Nicotinate and nicotinamide metabolism | -0.648030353 | Sig_Down |
| Porphyrin and chlorophyll metabolism | -1.024261231 | Sig_Down |
| Retinol metabolism | -2.407136684 | Sig_Down |
| Ubiquinone and other terpenoid-quinone biosynthesis | -1.079865012 | Sig_Down |
| Vitamin B6 metabolism | -0.382031379 | Sig_Down |
| Cyanoamino acid metabolism | -0.763832718 | Sig_Down |
| D-Glutamine and D-glutamate metabolism | -0.278686628 | Sig_Down |
| Glutathione metabolism | -0.661432613 | Sig_Down |
| Phosphonate and phosphinate metabolism | -1.169562964 | Sig_Down |
| Selenocompound metabolism | -0.616205762 | Sig_Down |
| Taurine and hypotaurine metabolism | -1.159634079 | Sig_Down |
| beta-Alanine metabolism | -2.911218578 | Sig_Down |
| Carotenoid biosynthesis | -3.454968488 | Sig_Down |
| Geraniol degradation | -5.220507565 | Sig_Down |
| Insect hormone biosynthesis | -2.969438471 | Sig_Down |
| Limonene and pinene degradation | -4.570547302 | Sig_Down |
| Nonribosomal peptide structures | -1.143165893 | Sig_Down |
| Terpenoid backbone biosynthesis | -0.463364975 | Sig_Down |
| Purine metabolism | -0.306764946 | Sig_Down |
| Aminobenzoate degradation | -2.656091438 | Sig_Down |
| Atrazine degradation | -4.385128455 | Sig_Down |
| Benzoate degradation | -3.095036867 | Sig_Down |
| Caprolactam degradation | -5.760833651 | Sig_Down |
| Chloroalkane and chloroalkene degradation | -1.991777391 | Sig_Down |
| Chlorocyclohexane and chlorobenzene degradation | -1.938885586 | Sig_Down |
| Dioxin degradation | -2.23561461 | Sig_Down |
| Drug metabolism - cytochrome P450 | -2.276617488 | Sig_Down |
| Ethylbenzene degradation | -5.434031495 | Sig_Down |
| Fluorobenzoate degradation | -4.784846992 | Sig_Down |
| Metabolism of xenobiotics by cytochrome P450 | -2.203700684 | Sig_Down |
| Naphthalene degradation | -1.782292968 | Sig_Down |
| Nitrotoluene degradation | -1.832723518 | Sig_Down |
| Polycyclic aromatic hydrocarbon degradation | -4.75024367 | Sig_Down |
| Steroid degradation | -9.896305919 | Sig_Down |
| Styrene degradation | -2.712453827 | Sig_Down |
| Toluene degradation | -4.694507698 | Sig_Down |
| Xylene degradation | -1.753123311 | Sig_Down |
| Longevity regulating pathway | -0.802504208 | Sig_Down |
| Longevity regulating pathway - multiple species | -1.311582687 | Sig_Down |
| Longevity regulating pathway - worm | -0.519402427 | Sig_Down |
| Adipocytokine signaling pathway | -1.867911779 | Sig_Down |
| Glucagon signaling pathway | -0.361239157 | Sig_Down |
| PPAR signaling pathway | -2.114350797 | Sig_Down |
| Renin-angiotensin system | -2.727690322 | Sig_Down |
| Thyroid hormone signaling pathway | -4.830751312 | Sig_Down |
| Plant-pathogen interaction | -0.728582776 | Sig_Down |
| Thermogenesis | -1.805023893 | Sig_Down |
| Proximal tubule bicarbonate reclamation | -1.38899837 | Sig_Down |
| Dopaminergic synapse | -5.537225859 | Sig_Down |
| GABAergic synapse | -1.09049115 | Sig_Down |
| Glutamatergic synapse | -0.961029436 | Sig_Down |
| Serotonergic synapse | -5.544286557 | Sig_Down |
| Autophagy - yeast | -1.290118486 | Sig_Down |
| MAPK signaling pathway - fly | -0.384759775 | Sig_Down |
| Prostate cancer | -1.271367215 | Sig_Down |
| Salmonella infection | -1.165635956 | Sig_Down |
| Tropane, piperidine and pyridine alkaloid biosynthesis | -0.832737823 | Sig_Down |
| Folate biosynthesis | -0.327921988 | Sig_Down |
| Pantothenate and CoA biosynthesis | -0.280401305 | Sig_Down |
| Biosynthesis of siderophore group nonribosomal peptides | -1.672440443 | Sig_Down |
| Estrogen signaling pathway | -1.271319754 | Sig_Down |
| Progesterone-mediated oocyte maturation | -1.271367215 | Sig_Down |
| Antigen processing and presentation | -1.271367215 | Sig_Down |
| IL-17 signaling pathway | -1.270995133 | Sig_Down |
| Th17 cell differentiation | -1.271367215 | Sig_Down |
| AMPK signaling pathway | -0.367918028 | Sig_Down |
| PI3K-Akt signaling pathway | -1.191538638 | Sig_Down |
| Renal cell carcinoma | -0.935348366 | Sig_Down |
| Cushing syndrome | -0.935348366 | Sig_Down |
| Epithelial cell signaling in Helicobacter pylori infection | -0.40413442 | Sig_Down |
| African trypanosomiasis | -1.322500277 | Sig_Down |
| Chagas disease (American trypanosomiasis) | -1.362972892 | Sig_Down |
| Huntington disease | -0.682222588 | Sig_Down |
| Glucosinolate biosynthesis | -0.30404417 | Sig_Down |
| Insulin signaling pathway | -0.469528633 | Sig_Down |
| NOD-like receptor signaling pathway | -0.504616108 | Sig_Down |
| Hematopoietic cell lineage | -11.39517217 | Sig_Down |
| C5-Branched dibasic acid metabolism | -0.389540194 | Sig_Down |
| Sphingolipid metabolism | -0.569673701 | Sig_Down |
| Riboflavin metabolism | -0.572349018 | Sig_Down |
| Isoquinoline alkaloid biosynthesis | -0.831284458 | Sig_Down |
| Polyketide sugar unit biosynthesis | -0.612603062 | Sig_Down |
| Mineral absorption | -0.911403881 | Sig_Down |
| Protein digestion and absorption | -0.968860962 | Sig_Down |
| Bacterial chemotaxis | -1.217909942 | Sig_Down |
| Biofilm formation - Pseudomonas aeruginosa | -0.549078965 | Sig_Down |
| Biofilm formation - Vibrio cholerae | -0.487563652 | Sig_Down |
| Phosphatidylinositol signaling system | -0.27305762 | Sig_Down |
| Biosynthesis of vancomycin group antibiotics | -0.50634165 | Sig_Down |
| Alzheimer disease | -0.298362536 | Sig_Down |
| Acarbose and validamycin biosynthesis | -0.492096279 | Sig_Down |
| Platinum drug resistance | -0.523634395 | Sig_Down |
| Apoptosis - multiple species | -1.127145006 | Sig_Down |
| p53 signaling pathway | -1.127145006 | Sig_Down |
| Colorectal cancer | -1.127145006 | Sig_Down |
| Small cell lung cancer | -1.125875902 | Sig_Down |
| Viral myocarditis | -1.127145006 | Sig_Down |
| Toxoplasmosis | -1.127145006 | Sig_Down |
| Hepatitis B | -1.127202156 | Sig_Down |
| Herpes simplex virus 1 infection | -1.127259304 | Sig_Down |
| Human cytomegalovirus infection | -1.125770923 | Sig_Down |
| Influenza A | -1.126829682 | Sig_Down |
| Kaposi sarcoma-associated herpesvirus infection | -1.125713774 | Sig_Down |
| Mannose type O-glycan biosynthesis | -1.361386705 | Sig_Down |
| Other types of O-glycan biosynthesis | -1.361386705 | Sig_Down |
| Non-alcoholic fatty liver disease (NAFLD) | -0.796562122 | Sig_Down |
| Parkinson disease | -0.794547039 | Sig_Down |

**Table S8.** Significant results for the tertiary KEGG functional pathways between the HM group and HN group.

| Pathway | log2(FC) | Regulation |
| --- | --- | --- |
| RNA transport | 0.36881181 | Sig_Up |
| Staphylococcus aureus infection | 0.555336866 | Sig_Up |
| Flavone and flavonol biosynthesis | 1.558848985 | Sig_Up |
| MicroRNAs in cancer | -0.458722218 | Sig_Down |
| Alcoholism | -1.129639233 | Sig_Down |
| Amphetamine addiction | -1.129639233 | Sig_Down |
| Cocaine addiction | -1.129639233 | Sig_Down |
| Lysine degradation | -0.84703786 | Sig_Down |
| Ascorbate and aldarate metabolism | -0.637777324 | Sig_Down |
| Butanoate metabolism | -0.690222311 | Sig_Down |
| Dopaminergic synapse | -1.12940926 | Sig_Down |
| GABAergic synapse | -0.529692269 | Sig_Down |
| Serotonergic synapse | -1.129492802 | Sig_Down |
| cAMP signaling pathway | -1.13560982 | Sig_Down |
| Caffeine metabolism | -1.137486362 | Sig_Down |
| Primary bile acid biosynthesis | -1.031075389 | Sig_Down |
| Steroid biosynthesis | -1.136394884 | Sig_Down |
| Steroid hormone biosynthesis | -1.134437956 | Sig_Down |
| Synthesis and degradation of ketone bodies | -0.908471912 | Sig_Down |
| Limonene and pinene degradation | -1.063905572 | Sig_Down |
| Atrazine degradation | -1.025328606 | Sig_Down |
| Caprolactam degradation | -1.112341365 | Sig_Down |
| Steroid degradation | -1.13853083 | Sig_Down |
| Thyroid hormone signaling pathway | -1.134388991 | Sig_Down |
| Hematopoietic cell lineage | -1.138687325 | Sig_Down |
| Glutamatergic synapse | -0.479376389 | Sig_Down |
| Meiosis - yeast | -0.96932205 | Sig_Down |
| Non-homologous end-joining | -1.101616281 | Sig_Down |
| Phenylalanine metabolism | -0.9609231 | Sig_Down |
| Tryptophan metabolism | -1.002356751 | Sig_Down |
| Valine, leucine and isoleucine degradation | -0.862881766 | Sig_Down |
| Fatty acid degradation | -0.929825524 | Sig_Down |
| beta-Alanine metabolism | -0.953995607 | Sig_Down |
| Geraniol degradation | -1.092500096 | Sig_Down |
| Insect hormone biosynthesis | -0.913563099 | Sig_Down |
| Benzoate degradation | -0.951808172 | Sig_Down |
| Fluorobenzoate degradation | -1.097483426 | Sig_Down |
| Toluene degradation | -1.061853676 | Sig_Down |
| PPAR signaling pathway | -0.813005441 | Sig_Down |
| Plant-pathogen interaction | -0.438889287 | Sig_Down |
| Ferroptosis | -0.682063814 | Sig_Down |
| Proteasome | -0.925570246 | Sig_Down |
| Protein processing in endoplasmic reticulum | -0.909157248 | Sig_Down |
| Type I diabetes mellitus | -0.265444978 | Sig_Down |
| Microbial metabolism in diverse environments | -0.487877871 | Sig_Down |
| Biosynthesis of unsaturated fatty acids | -0.716987882 | Sig_Down |
| Cyanoamino acid metabolism | -0.398323455 | Sig_Down |
| Ethylbenzene degradation | -1.098283766 | Sig_Down |
| Styrene degradation | -0.883149198 | Sig_Down |
| Adipocytokine signaling pathway | -0.786361143 | Sig_Down |
| Peroxisome | -0.672769676 | Sig_Down |
| Tyrosine metabolism | -0.747721583 | Sig_Down |
| Glyoxylate and dicarboxylate metabolism | -0.596444833 | Sig_Down |
| Degradation of aromatic compounds | -0.835571227 | Sig_Down |
| alpha-Linolenic acid metabolism | -1.065994742 | Sig_Down |
| Taurine and hypotaurine metabolism | -0.489235789 | Sig_Down |
| Chloroalkane and chloroalkene degradation | -0.71136083 | Sig_Down |
| Polycyclic aromatic hydrocarbon degradation | -1.056499621 | Sig_Down |
| Prostate cancer | -0.77264736 | Sig_Down |
| Alanine, aspartate and glutamate metabolism | -0.312626083 | Sig_Down |
| Arginine and proline metabolism | -0.536248162 | Sig_Down |
| Propanoate metabolism | -0.549019765 | Sig_Down |
| Fatty acid metabolism | -0.568046555 | Sig_Down |
| Retinol metabolism | -0.834443047 | Sig_Down |
| Aminobenzoate degradation | -0.867883444 | Sig_Down |
| Chlorocyclohexane and chlorobenzene degradation | -0.685546726 | Sig_Down |
| Dioxin degradation | -0.799719542 | Sig_Down |
| Longevity regulating pathway - multiple species | -0.579912803 | Sig_Down |
| Estrogen signaling pathway | -0.77264736 | Sig_Down |
| Progesterone-mediated oocyte maturation | -0.77264736 | Sig_Down |
| Antigen processing and presentation | -0.77264736 | Sig_Down |
| IL-17 signaling pathway | -0.772589609 | Sig_Down |
| Th17 cell differentiation | -0.77264736 | Sig_Down |
| Apoptosis - fly | -0.503031915 | Sig_Down |
| PI3K-Akt signaling pathway | -0.70115323 | Sig_Down |
| Pentose and glucuronate interconversions | -0.387198298 | Sig_Down |
| Nitrogen metabolism | -0.517613526 | Sig_Down |
| Carbon metabolism | -0.324142237 | Sig_Down |
| Selenocompound metabolism | -0.273069374 | Sig_Down |
| Xylene degradation | -0.664526636 | Sig_Down |
| Sphingolipid signaling pathway | -0.960029871 | Sig_Down |
| Glycine, serine and threonine metabolism | -0.283477575 | Sig_Down |
| Phenylpropanoid biosynthesis | -0.899869555 | Sig_Down |
| Inositol phosphate metabolism | -0.666719183 | Sig_Down |
| Methane metabolism | -0.327091162 | Sig_Down |
| Metabolic pathways | -0.27550168 | Sig_Down |
| Linoleic acid metabolism | -1.025123966 | Sig_Down |
| Longevity regulating pathway - worm | -0.264100862 | Sig_Down |
| Renin-angiotensin system | -0.95490868 | Sig_Down |
| Legionellosis | -0.302445595 | Sig_Down |
| Carbon fixation pathways in prokaryotes | -0.352006511 | Sig_Down |
| Sulfur metabolism | -0.616860389 | Sig_Down |
| Lipoarabinomannan (LAM) biosynthesis | -0.916182639 | Sig_Down |
| Nicotinate and nicotinamide metabolism | -0.333401367 | Sig_Down |
| Neomycin, kanamycin and gentamicin biosynthesis | -0.38473528 | Sig_Down |
| Pyruvate metabolism | -0.310365665 | Sig_Down |
| Biosynthesis of siderophore group nonribosomal peptides | -0.736574575 | Sig_Down |
| Carotenoid biosynthesis | -1.012647664 | Sig_Down |
| Drug metabolism - cytochrome P450 | -0.810901119 | Sig_Down |
| Chemical carcinogenesis | -0.92776443 | Sig_Down |
| Monobactam biosynthesis | -0.356429827 | Sig_Down |
| Biosynthesis of secondary metabolites | -0.265821487 | Sig_Down |
| Ether lipid metabolism | -0.979953416 | Sig_Down |
| Metabolism of xenobiotics by cytochrome P450 | -0.794634263 | Sig_Down |
| Necroptosis | -0.303377134 | Sig_Down |
| Bacterial chemotaxis | -0.738736393 | Sig_Down |
| Quorum sensing | -0.293461545 | Sig_Down |
| Basal transcription factors | -0.931453665 | Sig_Down |
| Arginine biosynthesis | -0.51518103 | Sig_Down |
| Naphthalene degradation | -0.659354148 | Sig_Down |
| Thermogenesis | -0.753785627 | Sig_Down |
| Proximal tubule bicarbonate reclamation | -0.630839111 | Sig_Down |
| Protein digestion and absorption | -0.990416507 | Sig_Down |
| Prodigiosin biosynthesis | -0.428052881 | Sig_Down |
| Citrate cycle (TCA cycle) | -0.440591564 | Sig_Down |
| Autophagy - yeast | -0.736178131 | Sig_Down |
| MAPK signaling pathway - yeast | -0.798341221 | Sig_Down |
| NOD-like receptor signaling pathway | -0.30968193 | Sig_Down |
| FoxO signaling pathway | -0.441765304 | Sig_Down |
| Porphyrin and chlorophyll metabolism | -0.539952172 | Sig_Down |
| Biosynthesis of terpenoids and steroids | -0.858652072 | Sig_Down |
| Oxidative phosphorylation | -0.387510008 | Sig_Down |
| Primary immunodeficiency | -0.273222118 | Sig_Down |
| Amyotrophic lateral sclerosis (ALS) | -0.800114218 | Sig_Down |
| Tropane, piperidine and pyridine alkaloid biosynthesis | -0.440556847 | Sig_Down |
| Phosphonate and phosphinate metabolism | -0.485367426 | Sig_Down |
| Longevity regulating pathway | -0.392653757 | Sig_Down |
| Bladder cancer | -0.976318341 | Sig_Down |
| Amoebiasis | -0.724207447 | Sig_Down |
| Apoptosis | -0.779421987 | Sig_Down |
| Two-component system | -0.295977992 | Sig_Down |
| Pathways in cancer | -0.587257933 | Sig_Down |
| Salmonella infection | -0.65523019 | Sig_Down |
| African trypanosomiasis | -0.631437097 | Sig_Down |
| Streptomycin biosynthesis | -0.405402357 | Sig_Down |
| Sphingolipid metabolism | -0.478568339 | Sig_Down |
| Nitrotoluene degradation | -0.642003097 | Sig_Down |
| Chagas disease (American trypanosomiasis) | -0.630622227 | Sig_Down |
| Novobiocin biosynthesis | -0.390493182 | Sig_Down |
| Secondary bile acid biosynthesis | -0.776655192 | Sig_Down |
| Flagellar assembly | -0.819608893 | Sig_Down |
| 2-Oxocarboxylic acid metabolism | -0.291978599 | Sig_Down |
| Histidine metabolism | -0.532801413 | Sig_Down |
| Fluid shear stress and atherosclerosis | -0.38161632 | Sig_Down |
| Prion diseases | -0.795601876 | Sig_Down |
| Valine, leucine and isoleucine biosynthesis | -0.273318082 | Sig_Down |
| Hepatocellular carcinoma | -0.605157238 | Sig_Down |
| Isoquinoline alkaloid biosynthesis | -0.534591486 | Sig_Down |
| Ubiquinone and other terpenoid-quinone biosynthesis | -0.463711543 | Sig_Down |
| Riboflavin metabolism | -0.329858052 | Sig_Down |
| Penicillin and cephalosporin biosynthesis | -0.461471697 | Sig_Down |
| Glutathione metabolism | -0.296492044 | Sig_Down |
| Apoptosis - multiple species | -0.822193831 | Sig_Down |
| p53 signaling pathway | -0.822193831 | Sig_Down |
| Colorectal cancer | -0.82219383 | Sig_Down |
| Small cell lung cancer | -0.821967 | Sig_Down |
| Viral myocarditis | -0.822193831 | Sig_Down |
| Toxoplasmosis | -0.822193831 | Sig_Down |
| Hepatitis B | -0.822214491 | Sig_Down |
| Herpes simplex virus 1 infection | -0.822235151 | Sig_Down |
| Human cytomegalovirus infection | -0.822024149 | Sig_Down |
| Influenza A | -0.822182837 | Sig_Down |
| Kaposi sarcoma-associated herpesvirus infection | -0.821967 | Sig_Down |
| Biotin metabolism | -0.297912906 | Sig_Down |
| Huntington disease | -0.333225476 | Sig_Down |
| Polyketide sugar unit biosynthesis | -0.359762018 | Sig_Down |
| Glycosaminoglycan degradation | -0.377334778 | Sig_Down |
| Betalain biosynthesis | -0.560149221 | Sig_Down |
| Nonribosomal peptide structures | -0.439056984 | Sig_Down |
| Furfural degradation | -1.243209427 | Sig_Down |
| Mineral absorption | -0.424429039 | Sig_Down |
| Biofilm formation - Pseudomonas aeruginosa | -0.281988468 | Sig_Down |
| Acarbose and validamycin biosynthesis | -0.284156014 | Sig_Down |
| Renal cell carcinoma | -0.348602377 | Sig_Down |
| Cushing syndrome | -0.348602377 | Sig_Down |
| RIG-I-like receptor signaling pathway | -0.313543588 | Sig_Down |
| Non-alcoholic fatty liver disease (NAFLD) | -0.422335422 | Sig_Down |
| Parkinson disease | -0.422229907 | Sig_Down |
| Mannose type O-glycan biosynthesis | -0.982803393 | Sig_Down |
| Other types of O-glycan biosynthesis | -0.982803393 | Sig_Down |

**Table S9.** Significant results for the tertiary KEGG functional pathways between the NM group and NN group.

| Pathway | log2(FC) | Regulation |
| --- | --- | --- |
| Dioxin degradation | 1.137159906 | Sig_Up |
| Naphthalene degradation | 0.825019432 | Sig_Up |
| Penicillin and cephalosporin biosynthesis | 0.694182365 | Sig_Up |
| Chlorocyclohexane and chlorobenzene degradation | 1.032147743 | Sig_Up |
| Xylene degradation | 0.898180957 | Sig_Up |
| Chloroalkane and chloroalkene degradation | 0.934819091 | Sig_Up |
| Glycosphingolipid biosynthesis - ganglio series | -1.076629224 | Sig_Down |
| Various types of N-glycan biosynthesis | -1.07664884 | Sig_Down |
| Biosynthesis of type II polyketide products | -3.500073599 | Sig_Down |
| C-type lectin receptor signaling pathway | -2.722466024 | Sig_Down |
| NF-kappa B signaling pathway | -2.552541023 | Sig_Down |
| TNF signaling pathway | -2.552541023 | Sig_Down |
| VEGF signaling pathway | -2.552541021 | Sig_Down |
| Leishmaniasis | -2.552541027 | Sig_Down |
| Regulation of lipolysis in adipocytes | -3.137503516 | Sig_Down |
| Glycosphingolipid biosynthesis - globo and isoglobo series | -1.074678783 | Sig_Down |
| Bisphenol degradation | -2.874469118 | Sig_Down |
| Fatty acid elongation | -2.66106548 | Sig_Down |
| Adherens junction | -10.9657843 | Sig_Down |
| Chemokine signaling pathway | -9.596550497 | Sig_Down |
| Retrograde endocannabinoid signaling | -2.415804683 | Sig_Down |
| Indole alkaloid biosynthesis | -2.327328081 | Sig_Down |
| Mannose type O-glycan biosynthesis | -0.975053643 | Sig_Down |
| Other types of O-glycan biosynthesis | -0.975053643 | Sig_Down |
| Renin secretion | -2.359895947 | Sig_Down |
| Staurosporine biosynthesis | -2.464765063 | Sig_Down |
| Furfural degradation | -2.322917032 | Sig_Down |
| Type I polyketide structures | -2.781359719 | Sig_Down |
| Pathogenic Escherichia coli infection | -4.137503524 | Sig_Down |
| Cell cycle | -1.396421821 | Sig_Down |
| Cell cycle - yeast | -1.472370677 | Sig_Down |
| Ovarian steroidogenesis | -1.552541021 | Sig_Down |
| Tetracycline biosynthesis | -3.459431624 | Sig_Down |
| Gap junction | -9.405069369 | Sig_Down |
| Biosynthesis of 12-, 14- and 16-membered macrolides | -11.9251423 | Sig_Down |
| Salivary secretion | -1.552541026 | Sig_Down |
| Synaptic vesicle cycle | -1.690044547 | Sig_Down |

**Table S10.** Metadata for the 16S rRNA sequencing samples used in this study.

| BioSample Name | SRA Sample Accession | Internal Sample ID | Group |
| --- | --- | --- | --- |
| YY-A711 | SAMN35578748 | YY11 | NM |
| YY-A713 | SAMN35578750 | YY13 | NM |
| YH-A742 | SAMN35578760 | YH2 | NM |
| YH-A743 | SAMN35578761 | YH3 | NM |
| YH-A745 | SAMN35578763 | YH5 | NM |
| YH-A754 | SAMN35578772 | YH14 | NM |
| YH-A756 | SAMN35578774 | YH16 | NM |
| YH-A758 | SAMN35578776 | YH18 | NM |
| YH-A759 | SAMN35578777 | YH19 | NM |
| YH-A761 | SAMN35578779 | YH21 | NM |
| YH-A762 | SAMN35578780 | YH22 | NM |
| BM-A771 | SAMN35578783 | BM1 | HM |
| BM-A775 | SAMN35578787 | BM5 | HM |
| BM-A776 | SAMN35578788 | BM6 | HM |
| BM-A777 | SAMN35578789 | BM7 | HM |
| BM-A782 | SAMN35578794 | BM12 | HM |
| BM-A795 | SAMN35578807 | BM25 | HM |
| BM-A797 | SAMN35578809 | BM27 | HM |
| BM-A800 | SAMN35578812 | BM30 | HM |
| BM-A805 | SAMN35578817 | BM35 | HM |
| BM-A817 | SAMN35578829 | BM47 | HM |
| BM-A819 | SAMN35578831 | BM49 | HM |
| BS-A823 | SAMN35578835 | BS1 | HM |
| BS-A825 | SAMN35578837 | BS3 | HM |
| BS-A826 | SAMN35578838 | BS4 | HM |
| BS-A829 | SAMN35578841 | BS7 | HM |
| BS-A835 | SAMN35578847 | BS13 | HM |
| BS-A853 | SAMN35578865 | BS29 | HM |
| BS-A858 | SAMN35578870 | BS34 | HM |
| YY-A703 | SAMN35578740 | YY3 | NN |
| YY-A705 | SAMN35578742 | YY5 | NN |
| YY-A717 | SAMN35578754 | YY17 | NN |
| YY-A706 | SAMN35578743 | YY6 | NN |
| YH-A741 | SAMN35578759 | YH1 | NN |
| BM-A778 | SAMN35578790 | BM8 | HN |
| BM-A779 | SAMN35578791 | BM9 | HN |
| BM-A783 | SAMN35578795 | BM13 | HN |
| BM-A784 | SAMN35578796 | BM14 | HN |
| BM-A799 | SAMN35578811 | BM29 | HN |
| BM-A808 | SAMN35578820 | BM38 | HN |
| BM-A815 | SAMN35578827 | BM45 | HN |
| BM-A816 | SAMN35578828 | BM46 | HN |
| BM-A821 | SAMN35578833 | BM51 | HN |
| BS-A824 | SAMN35578836 | BS2 | HN |
| BS-A830 | SAMN35578842 | BS8 | HN |
| BS-A832 | SAMN35578844 | BS10 | HN |
| BS-A840 | SAMN35578852 | BS18 | HN |
